# Mechanisms of pulmonary disease in F344 rats after workplace-relevant inhalation exposure to cross-linked water-soluble acrylic acid polymers

**DOI:** 10.1186/s12931-023-02355-z

**Published:** 2023-02-13

**Authors:** Shotaro Yamano, Tomoki Takeda, Yuko Goto, Shigeyuki Hirai, Yusuke Furukawa, Yoshinori Kikuchi, Kyohei Misumi, Masaaki Suzuki, Kenji Takanobu, Hideki Senoh, Misae Saito, Hitomi Kondo, Yoichiro Kobashi, Kenzo Okamoto, Takumi Kishimoto, Yumi Umeda

**Affiliations:** 1 Japan Bioassay Research Center, Japan Organization of Occupational Health and Safety, Hadano, Kanagawa 257-0015 Japan; 2 Department of Pathology, Tenri Hospital, Tenri, Nara 632-8552 Japan; 3 Department of Pathology, Hokkaido Chuo Rosai Hospital, Japan Organization of Occupational Health and Safety, Iwamizawa, Hokkaido 068-0004 Japan; 4 Director of Research and Training Center for Asbestos-Related Diseases, Okayama, Okayama 702-8055 Japan

**Keywords:** Cross-linked water-soluble acrylic acid polymer (CWAAP), Workplace-relevant inhalation exposure, Intratracheal instillation, Rat, Transforming growth factor, Pulmonary disease

## Abstract

**Background:**

Recently in Japan, six workers at a chemical plant that manufactures resins developed interstitial lung diseases after being involved in loading and packing cross-linked water-soluble acrylic acid polymers (CWAAPs). The present study focused on assessing lung damage in rats caused by workplace-relevant inhalation exposure to CWAAP and investigated the molecular and cellular mechanisms involved in lung lesion development.

**Methods:**

Using a whole-body inhalation exposure system, male F344 rats were exposed once to 40 or 100 mg/m^3^ of CWAAP-A for 4 h or to 15 or 40 mg/m^3^ of CWAAP-A for 4 h per day once per week for 2 months (9 exposures). In a separate set of experiments, male F344 rats were administered 1 mg/kg CWAAP-A or CWAAP-B by intratracheal instillation once every 2 weeks for 2 months (5 doses). Lung tissues, mediastinal lymph nodes, and bronchoalveolar lavage fluid were collected and subjected to biological and histopathological analyses.

**Results:**

A single 4-h exposure to CWAAP-A caused alveolar injury, and repeated exposures resulted in regenerative changes in the alveolar epithelium with activation of TGFβ signaling. During the recovery period after the last exposure, some alveolar lesions were partially healed, but other lesions developed into alveolitis with fibrous thickening of the alveolar septum. Rats administered CWAAP-A by intratracheal instillation developed qualitatively similar pulmonary pathology as rats exposed to CWAAP-A by inhalation. At 2 weeks after intratracheal instillation, rats administered CWAAP-B appeared to have a slightly higher degree of lung lesions compared to rats administered CWAAP-A, however, there was no difference in pulmonary lesions in the CWAAP-A and CWAAP-B exposed rats examined 18 weeks after administration of these materials.

**Conclusions:**

The present study reports our findings on the cellular and molecular mechanisms of pulmonary disease in rats after workplace-relevant inhalation exposure to CWAAP-A. This study also demonstrates that the lung pathogenesis of rats exposed to CWAAP-A by systemic inhalation was qualitatively similar to that of rats administered CWAAP-A by intratracheal instillation.

**Graphical Abstract:**

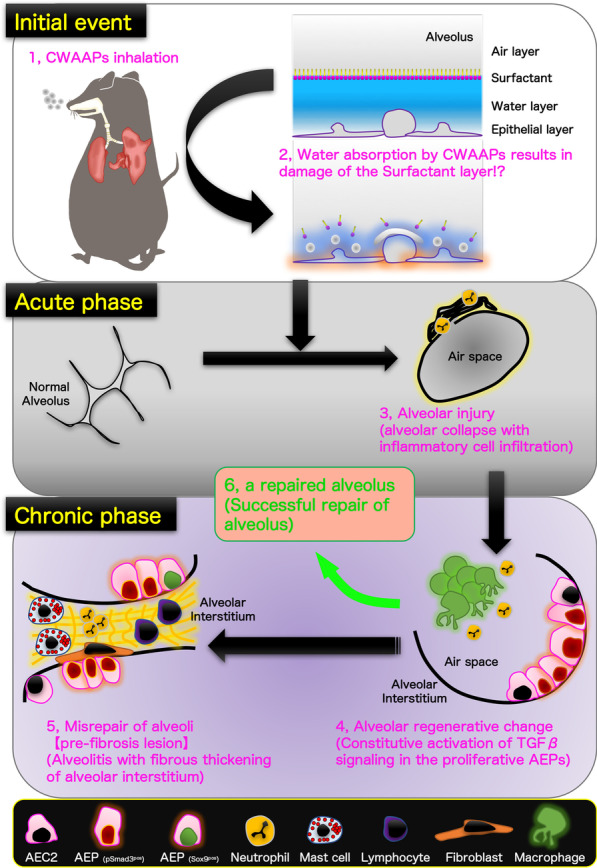

**Supplementary Information:**

The online version contains supplementary material available at 10.1186/s12931-023-02355-z.

## Background

Cross-linked water-soluble acrylic acid polymer (CWAAP) is a type of thickener that improves viscosity and sol–gel stability. CWAAPs have long been used in a variety of products, including cosmetics and pharmaceuticals, because of their low potential for skin and eye irritation. However, in Japan, six workers at a chemical plant that manufactures CWAAPs recently developed interstitial lung diseases after being involved in weighing, packing, and transporting CWAAP products [1]. Notably, five of these workers had a work history of only about 2 years. The 8-h weighted average (8 h-TWA) exposure to CWAAP for workers performing both loading and packaging was 3.2–7.6 mg/m^3^ with a maximum personal exposure to 41.8 mg/m^3^ during loading work (average time 22 min) [2]. Based on the results obtained from a clinical research project on this case [3, 4], in April 2019 the Ministry of Health, Labor and Welfare certified that five of the workers handling CWAAPs had incurred occupational injuries [5, 6].

We recently reported the results of a 13-week subchronic inhalation study that was conducted with reference to the OECD guidelines for the testing of chemicals, OECD TG 413 [7], to obtain reliable systemic toxicity data for occupational health [8]. Male and female F344 rats were exposed to 0, 0.3, 1, 3 and 10 mg/m^3^ CWAAP-A for 6 h per day, 5 days per week, for 13 weeks. Evaluation of all organs in the rats demonstrated that toxicity caused by inhalation exposure to CWAAP-A was limited to alveolar lesions in the lungs. Histopathological diagnosis of the lungs on the day after the final exposure showed that alveolar lesions, which were an inflammatory reaction, were observed in all rats exposed to 1, 3 and 10 mg/m^3^ CWAAP-A. The lowest observed adverse effect concentration (LOAEC) for CWAAP-A in this study was 1 mg/m^3^ for both sexes, and 0.3 mg/m^3^ was the no observed adverse effect concentration (NOAEC). It was also found that exposure to 3 and 10 mg/m^3^ CWAAP-A caused irreversible and progressive lung lesions [8]. Importantly, as mentioned above, workers were exposed for short time periods to extremely high levels of CWAAP, and the pulmonary toxic effects of such workplace-relevant inhalation exposure have not been studied. Therefore, in the present study, a high-concentration-intermittent-exposure protocol was adopted as being relevant to the exposure environment of workers at the CWAAP manufacturing site.

Interstitial lung disease encompasses numerous lung disorders characterized by inflammation and scarring (fibrosis). Pulmonary fibrosis destroys lung tissue and decreases elasticity, making it difficult for the lungs to get enough oxygen. It is well known that transforming growth factor (TGF) β signaling is important in the progression of pulmonary fibrosis [9]. TGFβ binding to its receptor activates a pathway that results in phosphorylation of the transcription factors Smad2 and Smad3, which form a heterotrimer with Smad4. This complex migrates to the nucleus where it induces the expression of a number of target genes that promote fibrosis [10]. Therefore, it is very important from a pathological viewpoint to clarify whether CWAAP affects TGFβ signaling and to identify the cells in which TGFβ signaling is activated. The possible involvement of alveolar epithelial progenitor cells (AEPs), which have recently attracted attention as a cell type activated during the repair process after lung injury [11, 12], is an important issue in CWAAP exposed lung pathology and in elucidating the mechanisms of disease progression. We have previously shown that AEPs are also present in rats [13]. Therefore, in the present study, we investigated the contribution of TGFβ signaling and AEPs in the development and progression of lung disease caused by exposure to CWAAPs.

Another important consideration is the large number of CWAAPs with different molecular weights and degrees of cross-linking and the lack of information on their hazard to human health. Thus, establishment of a rapid and simple method to evaluate the potential risk of different CWAAPs is also very important. Therefore, this study also investigated using intratracheal instillation as an adjunct to inhalation exposure.

## Methods

### Materials

CWAAP-A and CWAAP-B were purchased from a company which produces various polymers. The CWAAP-A was the same as the CWAAP-A used in our previous study [8]. As shown in Additional file 1: Fig. S1, CWAAP-A seems to be finer and lighter than CWAAP-B. A list of all primary antibodies used in these studies is summarized in Additional file 12: Table S1. The donkey anti-mouse IgG conjugated Alexa Fluor 488 (ab150105) and anti-rabbit IgG conjugated Alexa Fluor 594 (ab150064) were purchased from Abcam plc (Cambridge, UK). The VECTASHIELD Mounting Medium with DAPI (H-1200) was purchased from Vector laboratories (Burlingame, CA, USA). The other reagents were of the highest grade commercially available.

### Animals

All animal experiments were approved by the Animal Experiment Committee of the Japan Bioassay Research Center. Male F344 rats were purchased from Charles River Laboratories Japan, Inc. (Yokohama, Japan) and Japan SLC, Inc. (Hamamatsu, Japan). The rats were housed in an air-conditioned room under a 12 h light/12 h dark (8:00–20:00, light cycle) photoperiod, and fed a general diet (CRF-1, Oriental Yeast Co. Ltd., Tokyo, Japan) and tap water ad libitum. After 1–2 weeks of quarantine and acclimation, they were treated with CWAAPs.

### Whole body inhalation

In this study, a whole-body inhalation exposure protocol was used that was relevant to the exposure environment of workers at a CWAAP manufacturing site [1]. Two independent inhalation studies, repeated exposures and a single exposure, were conducted to clarify the molecular basis of lung lesions in the acute and chronic phases after CWAAP-A exposure (Additional file 2: Fig. S2). As the workplace concentrations of inhalable CWAAP were found to be above 40 mg/m^3^ in the field survey [2], a target concentration of 40 mg/m^3^ was used in both the repeated exposure study (15 and 40 mg/m^3^) and single exposure study (40 and 100 mg/m^3^) (see Additional file 2: Fig. S2A, B). OECD TG 433, an acute inhalation toxicity test guideline, states that a fixed duration of exposure of 4 h, excluding equilibration time, is recommended [14]. Based on this and the preliminary feasibility experiments (data not shown), the duration of exposure in both studies was 4 h. For the repeated exposure study, rats were exposed to CWAAP-A once a week for 8 weeks (9 exposures in total). The whole body inhalation was conducted using the direct-injection whole body inhalation system (Shibata Scientific Technology, Ltd., Soka, Japan) (Additional file 3: Fig. S3A). To prevent the CWAAP-A from absorbing moisture, the system was modified to supply dry air into the port of the cartridge. For the repeated exposure study, one inhalation chamber was used for exposure to sham air and two inhalation chambers were used for each target concentration of CWAAP-A to ensure that an adequate number of animals were exposed to each concentration of CWAAP-A (No.1 and 2: 15 mg/m^3^; and No. 3 and 4: 40 mg/m^3^). Inhalation exposure to CWAAP-A was started once the rats were 11 weeks old. A total 60 rats were used for the repeated exposure study. 6 rats in each group were sacrificed immediately after the last exposure and 6 rats in each group were sacrificed at the end of week 10. The remaining 8 rats in each group were sacrificed at the end of week 26 (see Additional file 2: Fig. S2A). A total of 24 rats were used for the single exposure study. 6 rats in the sham air group and 3 rats in the 40 and 100 mg/m^3^ groups were sacrificed 1 h after the end of the 4 h exposure period and the remaining rats were sacrificed 3 days after exposure (see Additional file 2: Fig. S2B).

Aerosol generation for CWAAP-A was conducted according to the instructions of equipment manufacturer. CWAAP-A was weighed and placed into the inner cartridge, which was then set into the dedicated port of the inhalation system. Compressed air was injected into the cartridge to generate an aerosol, which was then fed into the inhalation chamber. Compressed air at 0.4 Mpa was injected three times with a duration of 0.2 s and an interval of 0.3 s to empty the CWAAP-A from the cartridge. The interval between each series of injections was 4 min per cartridge (i.e., 60 cartridges were required for a 4-h exposure). The humidity (30–40%) in the chamber was adjusted using a valve linked to the humidification bottle. Once the humidity in the inhalation chamber was below 40%, the animals were placed in the chamber and exposure was initiated.

During exposure, the number of particles in each inhalation chamber was monitored by an optical particle controller (OPC-AP-600, Shibata Scientific Technology), and the concentration of CWAAP-A in the inhalation chamber was measured at least twice from hour 1 to hour 3 after the start of exposure by collecting the test substance in the inhalation chamber on a fluoropolymer binder glass fiber filter (TX40HI20-WW, φ55 mm, Tokyo Dylec, Corp., Tokyo, Japan). The particle size of CWAAP-A in the chamber was measured using a micro-orifice uniform deposit cascade impactor (MOUDI-II, MSP Corp., Shoreview, MN) during the second and the eighth exposures during the repeated exposure study. The mass median aerodynamic diameter (MMAD) and geometric standard deviation (σg) were calculated by cumulative frequency distribution graphs with logarithmic probability. In addition, CWAAP-A in the inhalation chamber was collected on a 0.2 μm polycarbonate filter (φ47 mm, Whatman plc, Little Chalfont, UK) and observed by scanning electron microscope (SEM) (SU8000, Hitachi High-Tech, Tokyo, Japan). Typical concentrations of CWAAP-A in the repeated exposure study are shown in Additional file 3: Fig. S3B. The measured concentrations of CWAAP-A in the chamber were maintained at approximately the target concentrations throughout the exposure period. The MMADs were 0.8 μm (Additional file 3: Fig. S3D, E) with σg values below 2.5 (Additional file 3: Fig. S3E). The coefficient of variation of the No.4 chamber was high because a system error that occurred due to the jamming of the cartridge in the cartridge holder during the seventh exposure. However, we confirmed that this error was resolved within one hour, and the average CWAAP-A concentration was maintained throughout the exposure period. The CWAAP-A particles generated in the chamber did not appear to be highly aggregated or humidified (Additional file 3: Fig. S3C). In the single exposure study, the measured concentrations of CWAAP-A were 42.5 mg/m^3^ in the 40 mg/m^3^ chamber and 100.4 mg/m^3^ in the 100 mg/m^3^ chamber, and the CWAAP-A MMAD and σg values were similar to those of the repeated exposure study. These data indicate that CWAAP-A exposure was stable throughout the exposure periods in both the repeated and single exposure studies.

In the repeated exposure study, rats were euthanized by exsanguination under isoflurane anesthesia immediately after the last exposure and at 2 weeks and 18 weeks after the last exposure. In the single exposure study, rats were sacrificed immediately after exposure and 3 days after exposure. BALF was collected as described below, and the lungs from which BALF was not collected were weighed and then fixed in 10% neutral phosphate-buffered formalin solution.

### Intratracheal instillation

CWAAP-A and CWAAP-B were suspended in PBS, sonicated and neutralized by 1 M NaOH. The final concentration of the CWAAPs solution was 1 mg/ml. Our preliminary study suggested that a single intratracheal administration of 1.5 mg/kg would have too strong an effect on the lung, while a dose of 0.5 mg/kg would be much weaker and return to normal in a few weeks (Additional file 4: Fig. S4). In addition, a previous study reported that a single intratracheal administration of CWAAPs at 0.1 mg/rat (about 0.43 mg/kg) caused a moderate inflammatory response [3]. Therefore, the dose of CWAAPs was set at 1 mg/kg, and the administration interval set at two weeks. 1 mg/kg corresponds to a 1-h exposure of a 60 kg worker (breathing rate: 1.2 m^3^/h) in an environment of 50 mg/m^3^, a level that can be considered relatively close to the short-term exposure to high concentrations that occurred at the CWAAP manufacturing site. For comparison with the whole body inhalation studies, CWAAPs were administered once every 2 weeks over an 8 week period (a total of 5 times) (Additional file 2: Fig. S2C). The Z-average (median particle diameter) and zeta potential of the preparations were measured using Zetasizer Ultra (Malvern Panalytical Ltd., Worcestershire, UK): CWAAP-A, 471 ± 136 nm and − 35.7 ± 1.7 mV; and CWAAP-B, 705 ± 53 nm and − 37.5 ± 2.5 mV. Intratracheal instillations of CWAAPs to rats was started when the rats were 8 weeks old. A total of 44 rats were used in the intratracheal instillation study (see Additional file 2: Fig. S2C). Rats were placed under isoflurane inhalation anesthesia and CWAAP solutions were injected at 1 mg/kg into the trachea of rats using a MicroSprayer^®^ Aerosolizer (Model IA-1B; Penn-Century, Inc., Wyndmoor, PA). At 2 weeks and 18 weeks after the final CWAAP administration, rats were placed under isoflurane anesthesia and euthanized by exsanguination. BALF was collected as described below. The left lung was then weighed and fixed in 10% neutral phosphate buffered formalin solution.

### BALF collection and analysis

The left (intratracheal instillation study and the single inhalation study) or right (repeated inhalation study) bronchus was tied off with a thread, and the opposite lung lobes were lavaged with 4–8 ml of saline. The total cell number in the BALF was counted using an Automated Hematology Analyzer (XN-2000 V; Sysmex Corp., Kobe, Japan). Cell populations on glass slides were prepared using Cytospin 4 (Thermo Fisher Scientific, Inc., Waltham, MA). After May-Grunwald-Giemsa staining, the differential white blood cell count was made by visual observation. To measure LDH activity, the BALF was centrifuged at 1960 rpm (800×*g*) for 10 min at 4 °C, and the supernatant was examined using an automatic analyzer (Hitachi 7080 or 7180, Hitachi High-Tech Corp., Tokyo, Japan).

### Enzyme immunoassay

SP-D concentrations in the BALF and plasma were determined using a Rat/Mouse SP-D kit YAMASA EIA (YAMASA Corp., Choshi, Japan). In this assay, the BALF was diluted 500- or 1,000-fold, and plasma was diluted 25-fold with the assay diluent included in the kit. BALF concentrations of TGFβ1 and TGFβ2 were measured by Human/Mouse/Rat/Porcine/Canine TGF-beta 1 Quantikine ELISA Kit (R&D Systems) and Mouse/Rat/Canine/Porcine TGF-beta 2 Quantikine ELISA Kit (R&D Systems). For the TGFβ2 assay, the BALF was diluted threefold with the sample diluent included in the kit. The absorbance at 450 nm was measured using a microplate reader (Spark^®^; Tecan Group, Ltd., Männedorf, Switzerland or SpectraMax; Molecular Devices, LLC., San Jose, CA, USA). For TGFβ1 and TGFβ2 assays, the absorbance at 570 nm was also measured and subtracted as the background signal.

### Hydroxyproline assay

The content of hydroxyproline, a main component of collagen, in the lungs was measured using a hydroxyproline assay kit (Perchlorate-Free) (BioVision, Inc., San Francisco, CA, USA). Small pieces of lung (about 30 mg) were homogenized in 10 volumes of distilled water using a portable power homogenizer (ASONE Corp., Osaka, Japan). The homogenates were processed according the kit's instructions. The hydroxyproline signal (absorbance at 560 nm) was measured using a microplate reader (Spark^®^; Tecan Group, Ltd., Männedorf, Switzerland or SpectraMax; Molecular Devices, LLC., San Jose, CA, USA).

### Histopathological analysis

Serial tissue sections were cut from paraffin-embedded lung specimens, and the first sections (2-μm thick) was stained with H&E for histological examination and the remaining sections were used for immunohistochemical analysis. The histopathological findings in this study for lung and mediastinal lymph node were determined after multifaceted discussions between certified pathologists from the Japanese Society of Toxicology and Pathology and certified medical pathologists from the Japanese Society of Pathology, based on the International Harmonization of Nomenclature and Diagnostic Criteria for Lesions in Rats and Mice (INHAND) [15]. Pathological diagnosis was performed blindly by three pathologists and summarized as a result of the discussion.

### Alcian blue staining

It is known that the alcian blue pH 1.0 method primarily detects sulfate groups, and the alcian blue pH 2.5 method preferentially detects carboxyl groups. CWAAPs were stained blue by both staining methods. The present study used the alcian blue pH 1.0 method, which did not stain the mucus of bronchial and alveolar epithelium (Additional file 5: Fig. S5). After deparaffinization and rinsing, the slides were incubated in 0.1 M HCl solution for 3 min. Then, they were incubated in alcian blue staining solution (alcian blue 8GX, C.I.74240, Merck-Millipore, Burlington, MA, USA) for 10 min at room temperature. The slides were then lightly passed through a 0.1N HCl solution and washed with flowing water. Finally, after contrast staining with Kernechtrot (NUCLEAR FAST RED, C.I.60760, Merck-Millipore) for 5 min, the slides were processed for light microscopy.

### Masson’s trichrome staining

The slides were deparaffinized, washed with water, and then reacted with a mixture of 10% potassium dichromate and 10% trichloroacetic acid for 60 min at room temperature. The specimens were then washed with water and stained with Weigert’s iron hematoxylin solution (C.I.75290, Merck-Millipore) for 10 min at room temperature. The slides were then successively stained with 0.8% orange G solution (C.I.16230, Merck-Millipore) for 10 min at room temperature, Ponceau (C.I.14700, FUJIFILM-Wako Pure Chemical Corp., Osaka, Japan) acid fuchsin (C.I.42685, Merck-Millipore) azophloxine (C.I.18050, Chroma Germany GmbH, Augsburg, Germany) mixture for 40 min at room temperature, 2.5% phosphotungstic acid for 10 min at room temperature, and blue aniline solution (C.I.42755, Chroma Germany GmbH) for 10 min at room temperature. Between each staining the slides were washed lightly with 1% acetic acid water. The slides were then processed for light microscopy.

### PAS-diastase staining

This method is a variant of PAS staining in which glycogen is degraded by pre-treatment with diastase to eliminate crossover against glycogen and improve specificity [16]. Slides were deparaffinized, rinsed with water, and digested with salivary amylase (in place of 1% diastase) at 37 °C for 60 min. The slides were then washed in water, reacted with 0.5% periodate for 10 min at room temperature, washed with distilled water, and reacted with Schiff reagent (Cold Schiff’s reagent, #40932, Muto Pure Chemicals Co. Ltd., Tokyo, Japan) for 30 min at room temperature. The reaction was stopped by washing three times with sulfurous acid water. The slides were then washed with water and stained with Mayer's Hematoxylin solution for 2 min at room temperature. After rinsing, the staining was checked under a microscope, and the slides were then processed for light microscopy.

### Immunohistological multiple staining analyses

Details of the multiple staining method have been described previously [17]. Briefly, lung tissue sections were deparaffinized with xylene, hydrated through a graded ethanol series, and incubated with 0.3% hydrogen peroxide for 10 min to block endogenous peroxidase activity. Slides were then incubated with 10% normal serum at room temperature (RT) for 10 min to block background staining, and then incubated for 2 h at RT with the first primary antibody. After washing with PBS, the slides were incubated with histofine simple stain ratMAX-PO(MULTI) (414191, Nichirei, Tokyo, Japan) for 30 min at RT. After washing with PBS, slides were incubated with DAB EqV Peroxidase Substrate Kit, ImmPACT (SK-4103, Vector laboratories) for 2–5 min at RT. Importantly, after washing with dH_2_O after color detection, the sections were treated with citrate buffer at 98 °C for 30 min before incubation with the next primary antibody to denature the antibodies already bound to the section. This procedure was repeated for the second and then the third primary antibodies. HighDef red IHC chromogen (ADI-950-142, Enzo Life Sciences, Inc., Farmingdale, NY, USA) was used for the second coloration and Histogreen chromogen (AYS-E109, Cosmo Bio, Tokyo, Japan) for the third coloration. Coloration was followed by hematoxylin staining for 30–45 s. The slides were then processed for light microscopy. For immunofluorescence staining, all primary and secondary antibodies used were made into a cocktail for each staining step and used simultaneously. After the fluorescence-labeled secondary antibodies reaction, the sections were shielded with DAPI-containing encapsulant and used for imaging. The sections were observed under an optical microscope ECLIPSE Ni (Nikon Corp., Tokyo, Japan) or BZ-X810 (Keyence, Osaka, Japan). For measurement of phospho-SMAD3 and Tm4sf1 positive indices, the sham air group (n = 5) and the 40 mg/m^3^ group immediately after exposure (n = 7), and the sham air group (n = 5) and the 40 mg/m^3^ group after an 18-week recovery period (n = 8) were used for analysis. For the 40 mg/m^3^ exposure group, positive indexes were counted separately for multifocal lesions and normal surrounding tissue. In all animals, at least five fields of view were measured using a 40 × objective lens. More than 500 cells per individual were measured for phospho-Smad3 and 300 cells per individual were measured for Tm4sf1, and the mean value per individual was used for statistical analysis.

### Statistical analysis

Except in the case of incidence and integrity of histopathological lesions, the data comparisons among multiple groups were performed by one-way analysis of variance with a post-hoc test (Dunnett’s or Tukey’s multiple comparison test), using GraphPad Prism 5 (GraphPad Software, San Diego, CA, USA). The incidences and integrity of lesions were analyzed by the chi-square test using GraphPad Prism 5 (GraphPad Software, San Diego, CA). All statistical significance was set at *p* < 0.05.

## Results

### Repeated systemic inhalation exposure to high concentrations of CWAAP-A caused pulmonary alveolar damage but not bronchiolar damage

**Fig. 1:**
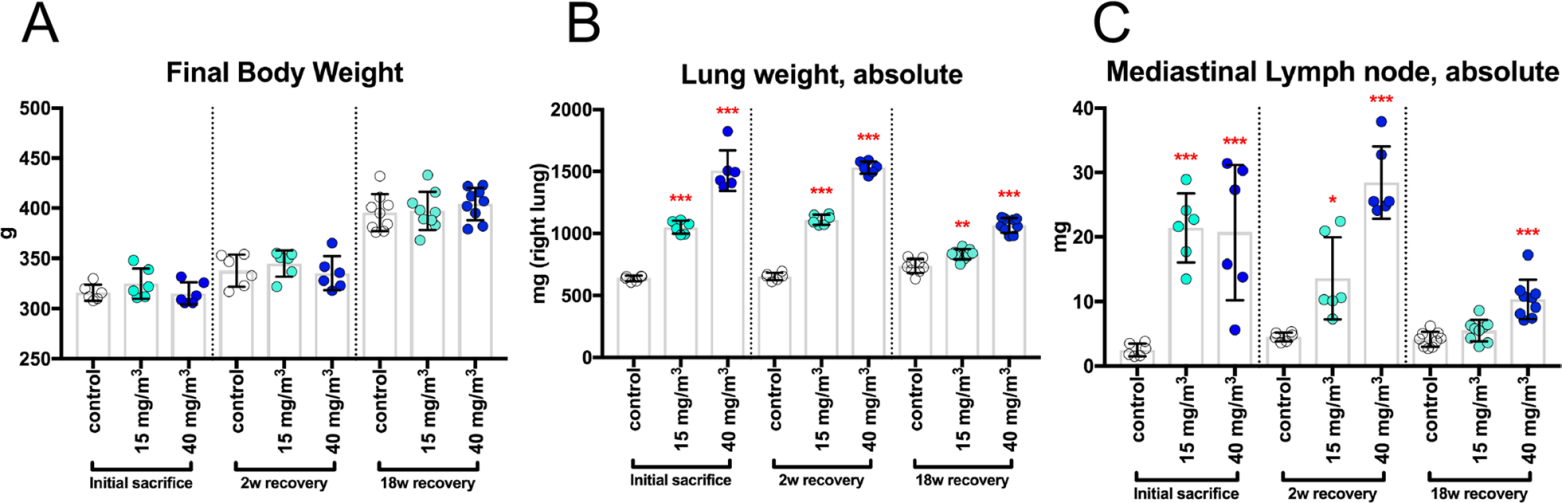
Final body weights and organ weights of the lung and mediastinal lymph nodes of male rats after repeated inhalation exposure to CWAAP-A. Results of the high-concentration intermittent inhalation experiment. Final body weights (**A**), right lung weights (**B**), and mediastinal lymph node weights (**C**) were measured at each sacrifice. Statistical significance was analyzed using Dunnett’s multiple comparison test compared with age-matched control (sham air) groups: *p < 0.05, **p < 0.01 and ***p < 0.001

Rats were exposed to CWAAP-A by inhalation at concentrations of 15 and 40 mg/m^3^ for 4 h per day once per week for 2 months (a total of 9 exposures): 40 mg/m^3^ is the highest exposure concentration found in the workplace. No significant effects on final body weight or general condition were observed (Fig. 1A). Immediately after exposure, there was a statistically significant, concentration-dependent increase in lung and mediastinal lymph node weights compared to the control group (Fig. 1B, C). These increases were still apparent after a 2-week recovery period (Fig. 1B, C). After 18 weeks recovery, the lung and mediastinal lymph node weights were less than the weights after the 2-week recovery period, but the lung weights were still significantly higher in both the 15 and 40 mg/m^3^ groups compared to the control group, and mediastinal lymph node weights were significantly higher in the 40 mg/m^3^ group compared to the control group (Fig. 1B, C).

**Fig. 2:**
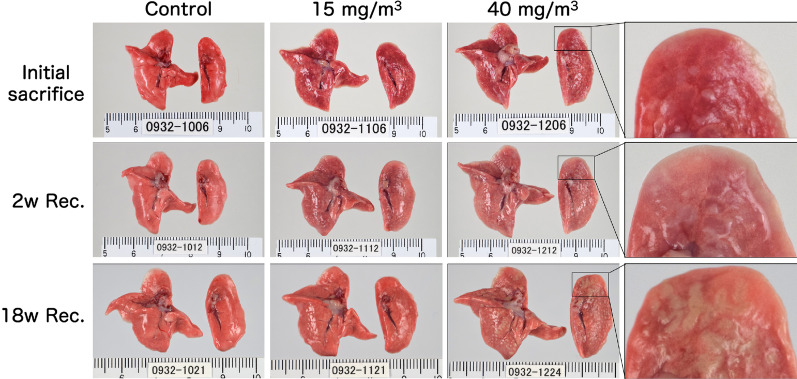
Representative macroscopic photographs of male rat lungs after repeated inhalation exposure to CWAAP-A. Results of the high-concentration intermittent inhalation experiment. High magnification views of the left lung are shown in the right panels. *Rec* recovery, *w* week

Representative images of the lungs are shown in Fig. 2. In the CWAAP-A-exposed groups, dark red patches, indicative of edema, were observed scattered over the surface of the lung immediately after the end of the exposure period and were still present after the 2-week recovery period (Fig. 2). After the 18-week recovery period, there was recovery of edematous changes, but white spots were observed throughout the lung. In addition to these white spots, distinctive white structures were observed on the cardiac surface of the left lung (Fig. 2, enlarged portion and Additional file 13: Table S2).

**Fig. 3:**
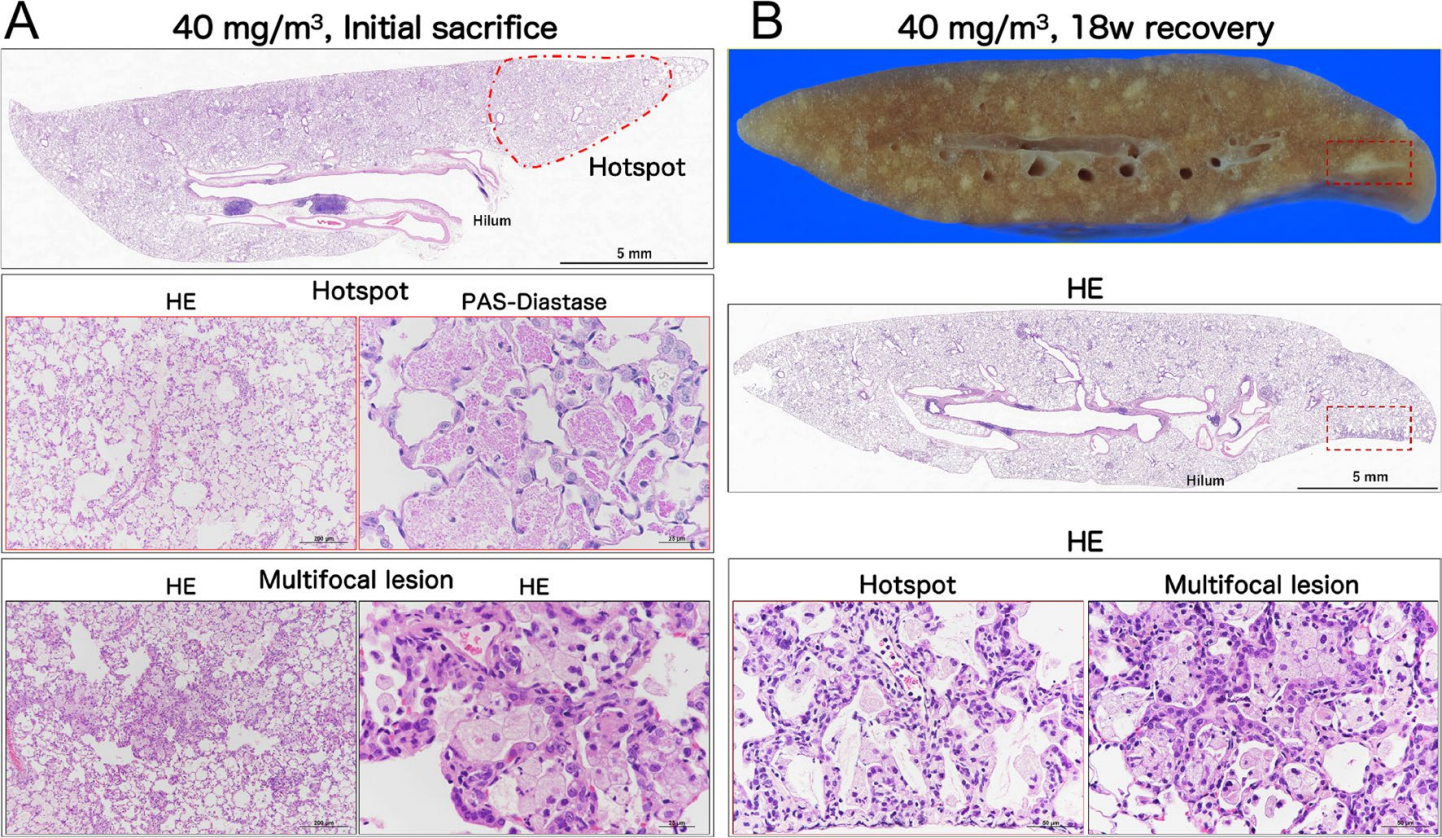
Representative macroscopic and microscopic photographs of rat lungs after repeated inhalation exposure to CWAAP-A. Results of the high-concentration intermittent inhalation experiment. Representative lesions of the rat lung exposed to 40 mg/m^3^ CWAAP-A are shown after hematoxylin and eosin (HE) and Periodic acid Schiff (PAS)-Diastase staining. The boxed areas in the upper and middle panels of 3B outline multifocal lesions after the 18-week recovery period

Representative histopathological photographs and cross-sectional images are shown in Additional file 6: Fig. S6 (control) and Fig. 3 (CWAAP-A). Figure 3A shows a representative lung section of a 40 mg/m^3^ exposed rat taken immediately after the end of the exposure period. There were areas of histopathologically observed alveolar proteinosis-like changes that could be identified by Periodic acid Schiff (PAS)-Diastase positive staining: in the area marked hotspot (Fig. 3A upper panel), PAS-Diastase staining was positive (Fig. 3A middle panel), indicating the presence of lipoprotein-like material, which is reminiscent of pulmonary alveolar proteinosis. In addition, multifocal lesions consisting of hypertrophy/proliferation of alveolar epithelium and inflammation were observed as white spots (Fig. 3A lower panel). Hotspot PAS-Diastase positive staining and multifocal lesions (white spots) were also observed in the lungs of rats exposed to 15 mg/m^3^ CWAAP-A.

Figure 3B shows a representative lung section of a 40 mg/m^3^ exposed rat taken after the 18-week recovery period. Prominent white spots (multifocal lesions) were observed in lung cross-sections (B, upper and middle panels). In the hotspot area, a large number of inflammatory cells were observed in the alveolar and perivascular interstitium, spreading to the subpleural area (B, middle and lower left): we diagnosed this as alveolitis. Alveolitis was also observed in multifocal lesions (B, lower right). Alveolitis also developed in the lungs of rats exposed to 15 mg/m^3^ CWAAP-A. These findings indicate that the lesions observed at the end of the exposure period in rats exposed to 15 and 40 mg/m^3^ CWAAP-A continued to develop during the recovery period. In sharp contrast to the alveoli, in the bronchus and bronchiole regions neither epithelial cells nor the surrounding interstitium were prominently affected by CWAAP-A exposure (Additional file 7: Fig. S7)

**Fig. 4:**
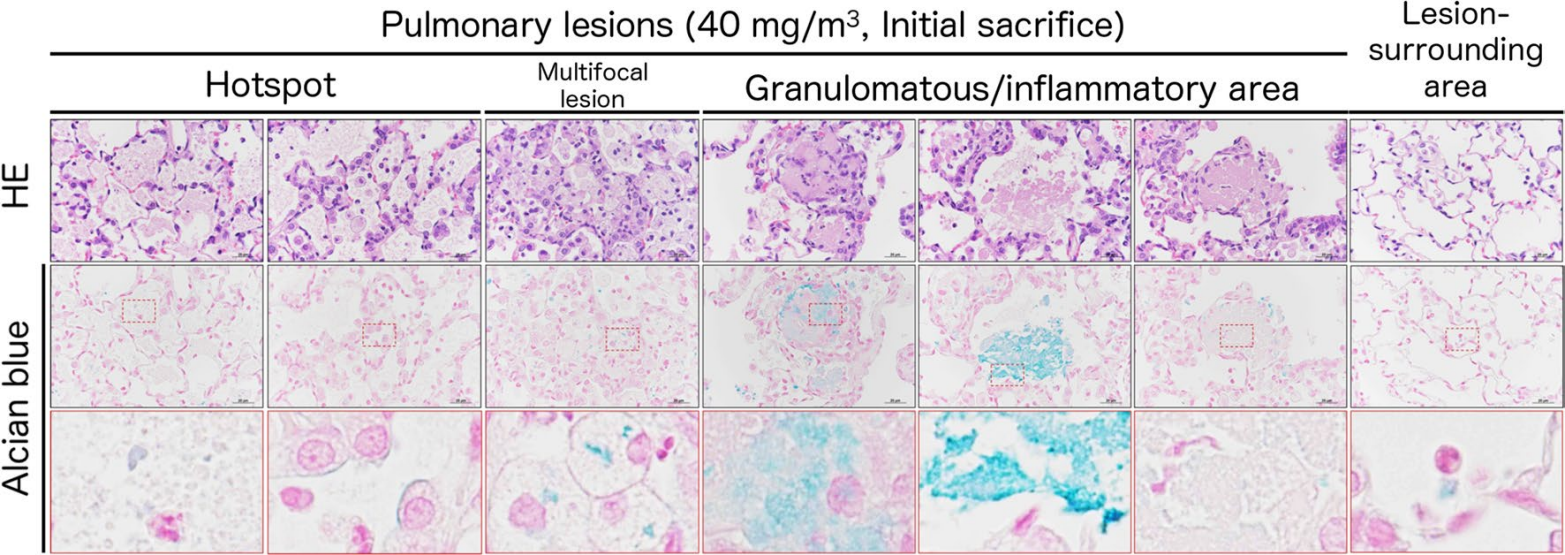
Representative images of alcian blue staining together with HE staining in the lesions and lesion-surrounding tissues of the rat lungs after repeated inhalation exposure to 40 mg/m^3^ CWAAP-A. All data are the results of the high-concentration intermittent inhalation experiment

CWAAPs have numerous carboxyl groups (Additional file 1: Fig. S1); therefore, to observe the localization of CWAAP-A in lung tissue we investigated staining methods that in principle react with carboxyl groups. Consequently, we focused on a modified alcian blue staining method, and found a condition in which the mucus of epithelial cells in normal rat lung tissue was not stained, while CWAAP-A particles were stained blue (Additional file 5: Fig. S5). The results of representative alcian blue staining and hematoxylin and eosin (HE) staining in serial sections of a rat lung from the 40 mg/m^3^ CWAAP-A exposed group immediately after the exposure period are shown in Fig. 4. In the lungs of the CWAAP-A-exposed group, blue-stained areas were observed on the apical cell membranes of the alveolar epithelium, in the alveolar air space, and in the macrophage cytoplasm. The positive areas were not localized to foci. Within the granulomatous lesions in the alveolar region, prominent blue areas could be observed, but alcian blue staining varied considerably between lesions. These results suggest that alcian blue staining may be useful for the observation of intrapulmonary CWAAPs. However, further development of the method is necessary to fully understand the localization of CWAAPs.

**Fig. 5:**
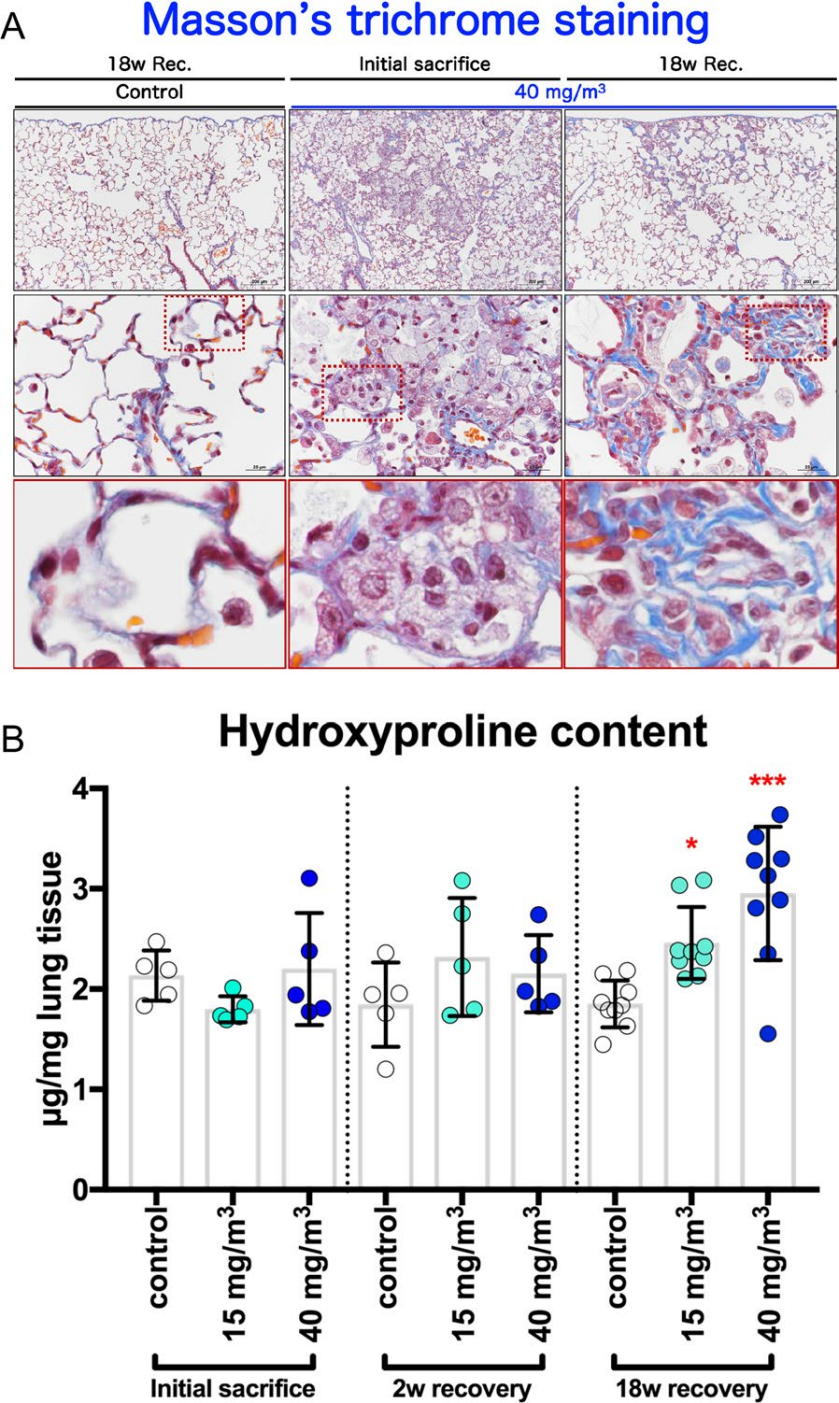
Collagen deposition of the rat lungs after repeated inhalation exposure to CWAAP-A. Results of the high-concentration intermittent inhalation experiment. Representative images of Masson’s trichrome staining (**A**) and hydroxyproline content (**B**) in the lung. Dunnett’s multiple comparison test of rats exposed to CWAAP-A with age-matched control (sham air) groups shows a significant increase in hydroxyproline content after the 18 week recovery period: *p < 0.05 and ***p < 0.001

To examine changes in collagen fiber volume, Masson's trichrome staining and the measurement of hydroxyproline levels in the lungs were performed. The results showed that in the lungs of the 40 mg/m^3^ exposure group after the 18-week recovery period there was a marked increase in Masson stain-positive areas in the alveolar septa and perivascular interstitium within the alveolitis (Fig. 5A right panels) compared to the controls and to the rat lung at the end of the exposure period (Fig. 5A left and middle panels). Consistent with this, the hydroxyproline content in the lungs showed significant increases over controls only in the exposed groups sacrificed after the 18-week recovery period (Fig. 5B): there was a concentration-dependent increase in hydroxyproline content in the lungs. These results indicate that prominent fibrous thickening (an increase in collagenous fibers) in the interstitium progressed over time.

The histopathological findings in the lung and mediastinal lymph nodes are shown in Table 1. The enlargement of the mediastinal lymph nodes observed in the exposed groups was histopathologically diagnosed as lymphoid hyperplasia. The rats recovered from this condition over time, and no lymphoid hyperplasia was observed after the 18-week recovery period. In the lung, granulomatous change, multifocal lesions, and accumulation of lipoproteinous material were observed in the alveolar region in the exposed groups. The multifocal lesions consisted of inflammation in the air space (similar to lipoid pneumonia) and hypertrophy/proliferation of alveolar epithelium in the animals immediately after exposure. In addition, after the 18-week recovery period, histopathological findings included cholesterol cleft in the air space (also known as cholesterol granuloma), alveolitis (also known as interstitial pneumonia), and fibrous thickening in the interstitium.

**Table 1:** Histopathological findings of the mediastinal lymph node and lung after repeated inhalation exposure to CWAAP-A

Experimental weeks	8 w			2 w recovery			18 w recovery		
Exposure concentration (mg/m^3^)	0	15	40	0	15	40	0	15	40
No. of animals examined	6	6	6	6	6	6	9	9	10
Histopathological findings									
Mediastinal lymph node									
Lymphoid hyperplasia	0	3 *	4 *	0	4 *	3 *	0	0	0
		< 1 >	< 1.5 >		< 1.3 >	< 2 >			
Lung									
Granulomatous change, alveolar	0	6 ***	6 ***	0	6 ***	6 ***	0	9 ***	10 ***
		< 1 >	< 2.2 >		< 1 >	< 2 >		< 1 >	< 1 >
Multifocal lesion, alveolar									
Hypertrophy/proliferation of alveolar epithelium	0	6 ***	6 ***	0	6 ***	6 ***	0	8 ***	10 ***
		< 1.2 >	< 3 >		< 1 >	< 2 >		< 1 >	< 2 >
Inflammation, air space	0	6 ***	6 ***	0	6 ***	6 ***	0	8 ***	10 ***
		< 1.2 >	< 3 >		< 1 >	< 2 >		< 1 >	< 1 >
Cholesterol cleft, air space	0	0	0	0	0	0	0	3	10 ***
								< 1 >	< 1 >
Alveolitis	0	0	0	0	0	0	0	3	10 ***
								< 1 >	< 2 >
Fibrous thickening, interstitial	0	0	0	0	0	0	0	0	10 ***
									< 1 >
Accumulation of lipoproteinous material, air space	0	5 **	6 ***	0	2	6 ***	0	0	0
		< 1 >	< 2 >		< 1 >	< 1.2 >			
Hyperplasia, bronchiolo-alveolar	0	0	0	0	0	0	0	0	1
									< 1 >

Values indicate number of animals bearing lesions

The value in angle bracket indicate the average severity grade index of the lesion. The average severity grade is calculated using the following equation: Σ(grade × number of animals with grade)/number of affected animals

Grade: 1, slight; 2, moderate; 3, marked; 4, severe. Significant difference: *p < 0.05; **p < 0.01; ***p < 0.001 by Chi square test compared with the respective controls

Interestingly, after the 18-week recovery period, one animal in the 40 mg/m^3^ group showed bronchiolo-alveolar hyperplasia, a pre-neoplastic lesion. Although this lesion was found in only one of the ten animals and is consequently without statistical significance, it was too early to appear as an age-related lesion, suggesting that it may have been caused by CWAAP-A exposure.

**Fig. 6:**
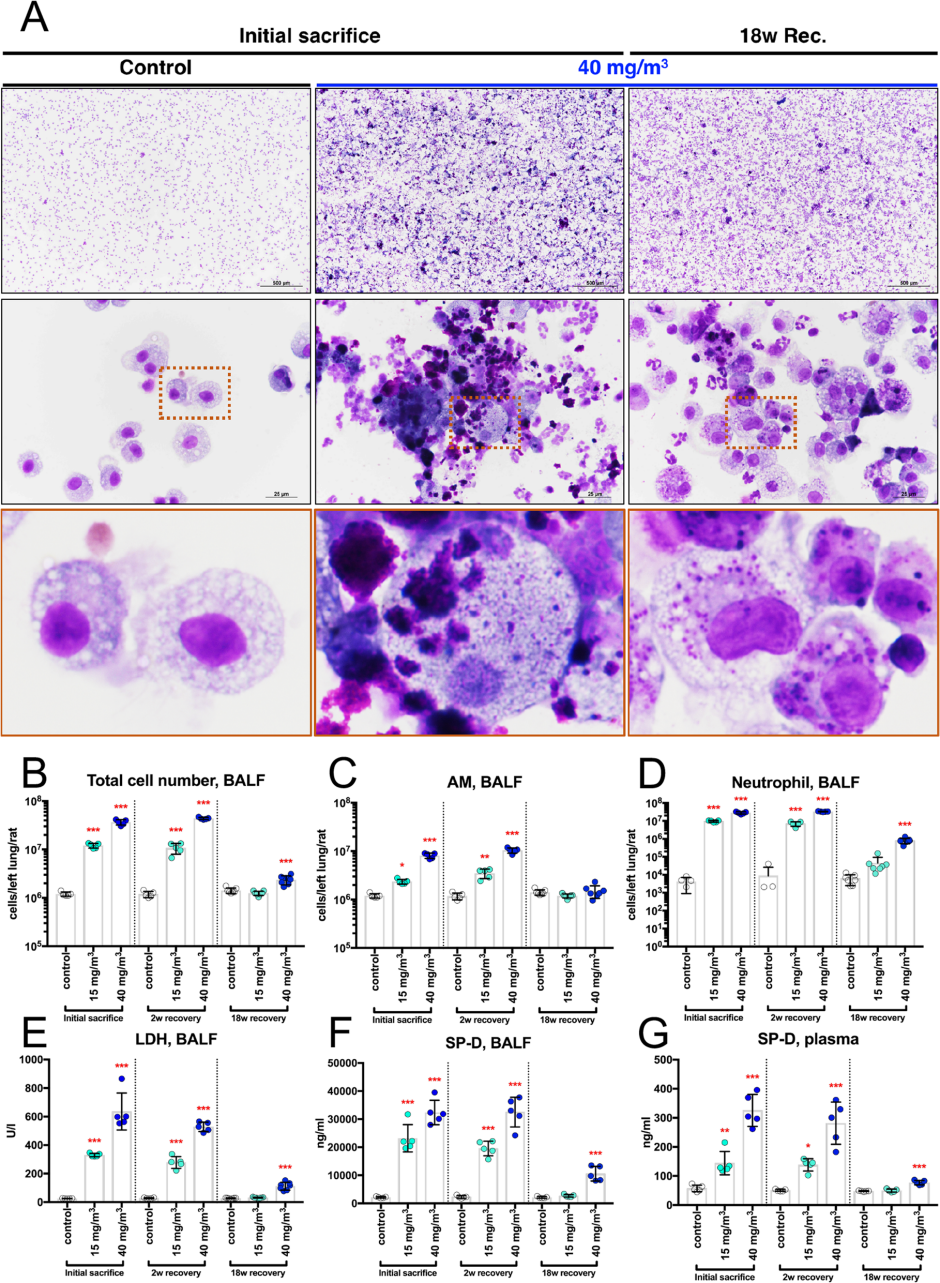
Representative images of the bronchoalveolar lavage fluid (BALF) cytospin cytology. Results of the high-concentration intermittent inhalation experiment. BALF cytospin cytology (**A**). Total cell number (**B**), alveolar macrophage (AM) number (**C**), neutrophil number (**D**), LDH activity (**E**), and surfactant protein-D (SP-D) level (**F**) in the BALF and SP-D level in the plasma (**G**). Statistical significance was analyzed using Dunnett’s multiple comparison test: *p < 0.05, **p < 0.01, and ***p < 0.001 versus controls

Figure 6 shows the results of the analysis of bronchoalveolar lavage fluid (BALF). In the sham air group, normal macrophages with fine vacuoles were observed. However, in the 40 mg/m^3^ group, a large number of neutrophils, CWAAP-A deposits, and enlarged macrophages phagocytosing CWAAP-A were observed both immediately after the exposure period and after the 18-week recovery period (Fig. 6A). The cell numbers found in the BALF are shown in Fig. 6B–D. Lactate dehydrogenase (LDH) activity, a cytotoxicity marker, and surfactant protein-D (SP-D) levels, an interstitial pneumonia marker [18], in the BALF and the plasma are shown in Fig. 6E–G. Immediately after exposure, all of these markers were significantly increased compared to the control group in a concentration-dependent manner. This increase was maintained after the 2-week recovery period. However, after the 18-week recovery period, there was a marked decrease in these marker values compared to the values immediately after exposure, and the 15 mg/m^3^ group recovered to the same level as the control group. These results support the results obtained from histopathological examinations that CWAAP-A caused a multifocal pattern of alveolar lesions, including inflammation in the air space.

These results indicate that repeated inhalation exposure to high concentrations of CWAAP-A cause pulmonary alveolar but not bronchiolar damage in the rat. In rats exposed to CWAAP-A at 40 mg/m^3^, there was a multifocal pattern of alveolar lesions that developed into interstitial alveolar lesions with collagen deposition.

### Continuous activation of TGFβ signaling in AEC2 by CWAAP-A exposure contributes to the progression of rat pulmonary disorders

**Fig. 7:**
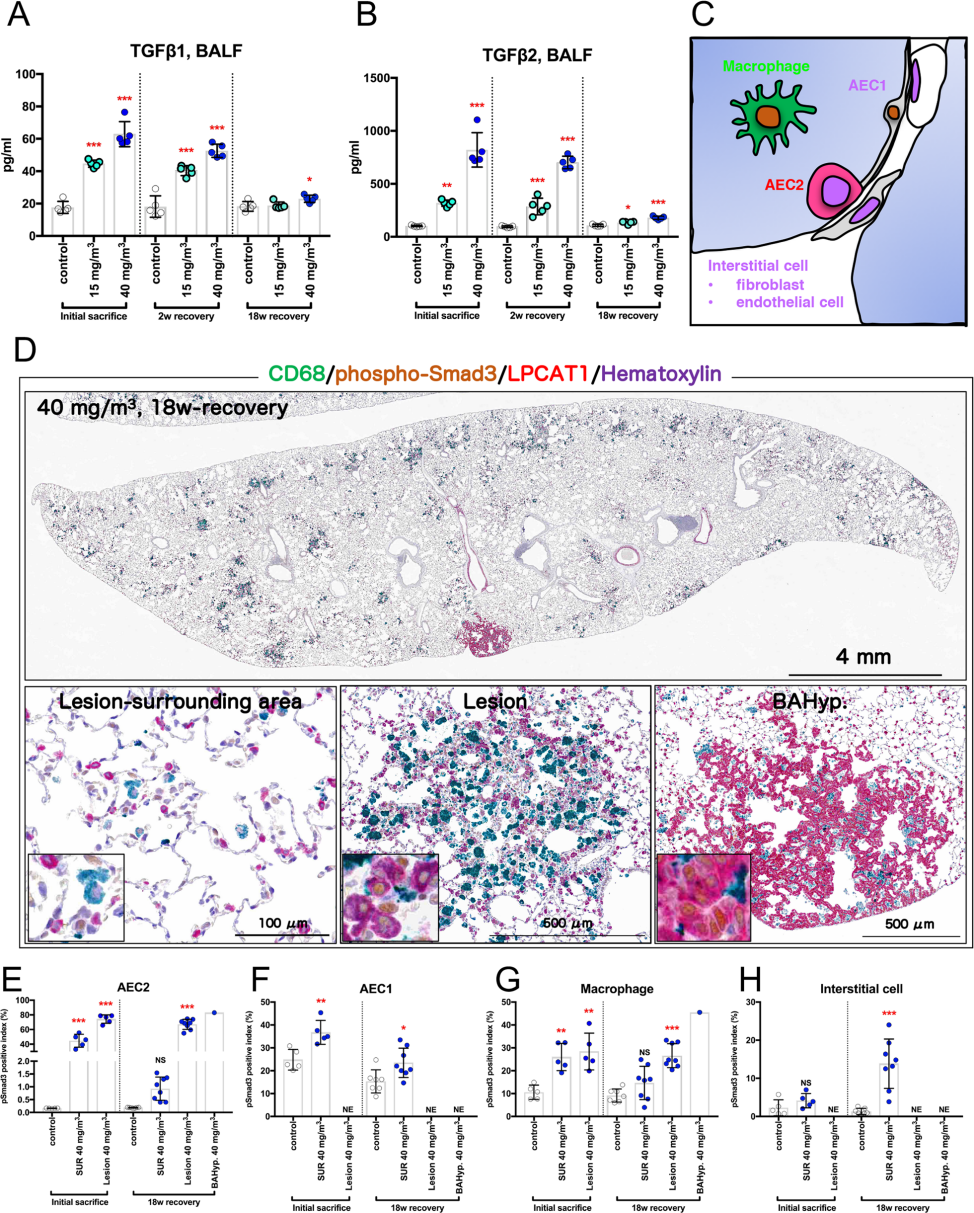
Examination of transforming growth factor (TGF) β signaling in the lung. Results of the high-concentration intermittent inhalation experiment. The level of TGFβ1 (**A**) and TGFβ2 (**B**) in the BALF are shown. Schematic diagram of the alveoli after CD68-phospho-Smad3-lysophosphatidylcholine acyltransferase 1 (LPCAT1) triple staining (**C**): macrophages and alveolar epithelial type 2 cells (AEC2) were stained green and red, respectively, and alveolar epithelial type 1 cells (AEC1) and interstitial cells, including both fibroblast and endothelial cells, were not stained. Representative immunohistochemical staining images are shown in panel **D**. Nuclear phospho-Smad3 positive indexes of AEC2 (**E**), AEC1 (**F**), macrophage (**G**) and interstitial cells (**H**) are shown as bar graphs. Statistical significance was analyzed using Dunnett’s multiple comparison test: **p < 0.01 and ***p < 0.001 versus controls. *NE* not examined, *NS* not significant, *SUR* lesion-surrounding tissues, *BAHyp* bronchiolo-alveolar hyperplasia

TGFβ signaling is known to contribute to the pathogenesis and progression of fibrosis across multiple organs [9]. The results shown in the previous section indicate that inhalation exposure to CWAAP-A results in alveolitis with fibrous thickening of the interstitium. Therefore, we focused on TGFβ signaling to investigate the mechanism of CWAAP-A induction of lung lesions in rats. The TGFβ1 and TGFβ2 levels in the BALF are shown in Fig. 7A, B, respectively. Immediately after exposure and after the 2-week recovery period, both TGFβ ligands showed a significant concentration-dependent increase in the exposed groups compared to the control group. After the 18-week recovery period, the induced levels of TGFβ had dropped considerably compared to the immediate post-exposure period, however, the 40 mg/m^3^ group still showed a small but significant increase in both TGFβ1 and TGFβ2 levels compared to the control group.

Immunostaining with antibodies that recognize Smad3 phosphorylated at serine residues S423 and S425, lysophosphatidylcholine acyltransferase 1 (LPCAT1; an alveolar epithelial type 2 cell (AEC2) marker), and CD68 (ED-1; a macrophage marker) was used to identify the cell types in which TGFβ signaling was activated. The TGFβ signaling responder cells show brown nuclei (phospho-Smad3-positive), macrophages show green cytoplasm (CD68-positive), and AEC2 cells show red cytoplasm (LPCAT1-positive) (Fig. 7C). The alveolar epithelial type 1 cell (AEC1) has a nucleus protruding into the alveolar space, and alveolar interstitial cells are mainly vascular endothelial cells and fibroblasts in the alveolar interstitium, which are all CD68-LPCAT1 double negative (Fig. 7C). AEC2 were mainly visualized in bronchiolo-alveolar hyperplasia, while AEC2 and macrophages were mixed in multifocal lesions in agreement with the pathological morphology (Fig. 7D). In normal alveolar tissue, the cell type with the lowest phospho-Smad3 positivity was AEC2 (Fig. 7E). However, the nuclear expression of phospho-Smad3 was most markedly increased in AEC2 immediately after the end of the CWAAP-A exposure period. This high level of phospho-Smad3 positive AEC2 was seen in both the lesions and in the surrounding tissues (Fig. 7E–H), with the positive index in the lesion reaching approximately 80% (Fig. 7E). Interestingly, after the 18-week recovery period, the foci in the lungs of rats in the 40 mg/m^3^ group continued to show a marked increase of phospho-Smad3 positivity in AEC2, while the significant increase in the surrounding tissues disappeared (Fig. 7E). Furthermore, AEC2 in bronchiolo-alveolar hyperplasia (BAHyp) and AEC2 in multifocal lesions were found to be highly positive for phospho-Smad3 (Fig. 7D–H). These results indicate that CWAAP-A increases TGFβ ligands in the lung and that TGFβ signaling is markedly elevated, especially in the AEC2 cells in CWAAP-A induced lesions. Furthermore, data after the 18-week recovery period showed that TGFβ signaling was still markedly elevated in AEC2 cells within the lesion despite a marked decrease in lung TGFβ ligand levels. The continuous activation of TGFβ signaling in AEC2 cells within the lesion may play an essential role in the progression to fibrotic interstitial lesions.

### Alveolar epithelial progenitor cells (AEPs) are conserved in the rat lung, and expansion of AEPs are responsible for the progression of CWAAP-A induced pulmonary disorders

**Fig. 8:**
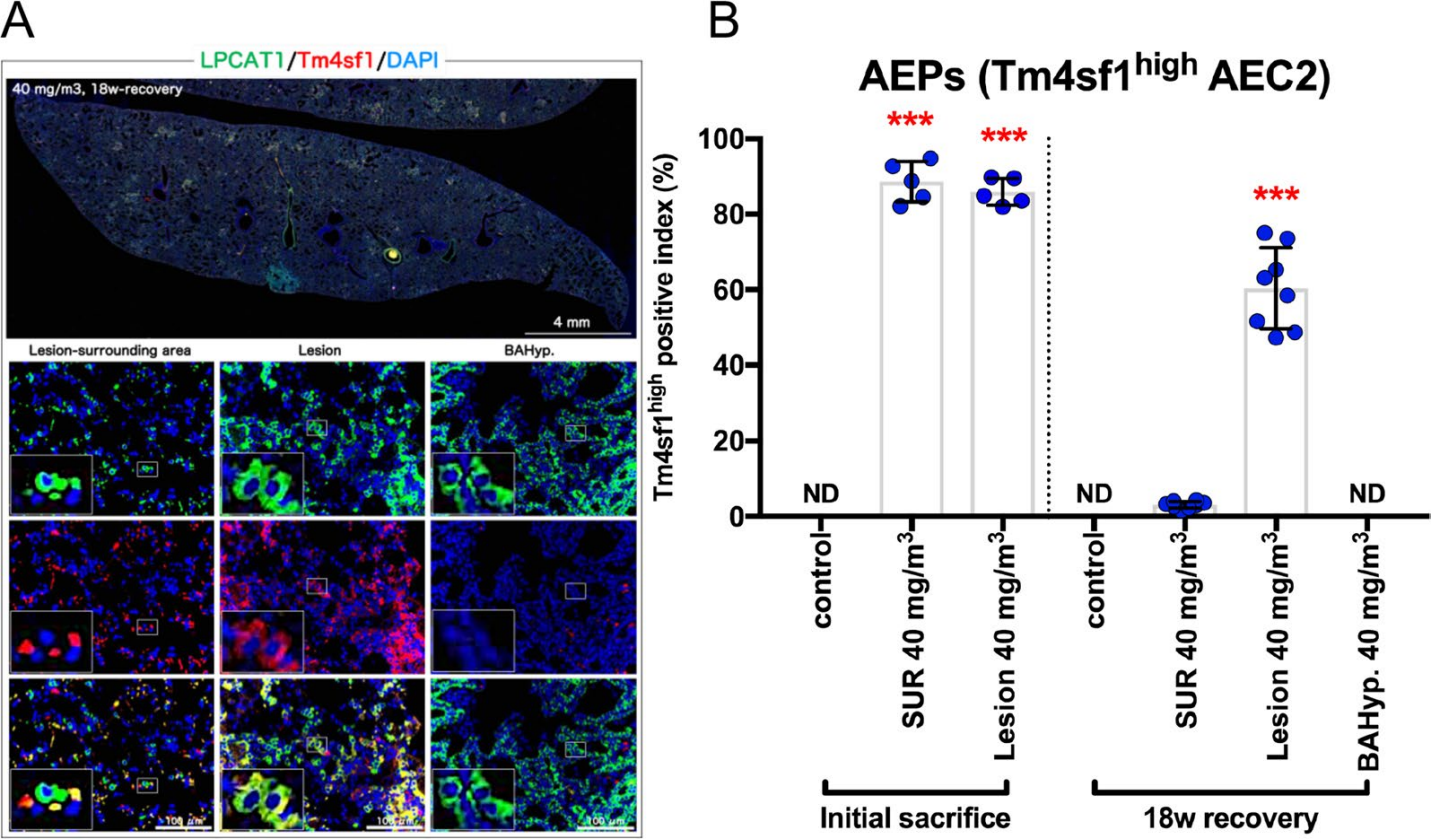
Examination of alveolar epithelial progenitor cells (AEPs) in the lung. Results of the high-concentration intermittent inhalation experiment. Representative images of LPCAT1 (an AEC2 marker, green), transmembrane 4 L six family member 1 (Tm4sf1) (an AEP marker, red), and 4′,6-diamino-2-phenylindole (DAPI) (a nucleus marker, blue) co-staining (**A**). The Tm4sf1-high positive index was measured (**B**). Statistical significance was analyzed using Dunnett’s multiple comparison test: ***p < 0.001 versus controls. *ND* not detectable

As described above, in rats exposed to CWAAP-A, alveolar lesions centered on AEC2 were consistently observed from immediately after exposure to the end of the 18-week recovery period. Recently, it has been reported that AEPs exist in mice and humans, and play an important role in alveolar regeneration [11, 12]. We investigated the hypothesis that AEPs may be involved in CWAAP-A induced rat lung lesions. Based on the report by Zacharias et al. that transmembrane 4 L six family member 1 (Tm4sf1) is an AEP marker [11], we performed immunohistochemistry for Tm4sf1. We found that alveolar epithelium highly expressing Tm4sf1 was only present in CWAAP-A induced lung lesions (Additional file 8: Fig. S8, middle panels). Interestingly, the expression of Tm4sf1 in bronchiolo-alveolar hyperplasia is as low as that in the surrounding tissues (Additional file 8: Fig. S8, left panel, right panel), suggesting specificity of high Tm4sf1 expression in CWAAP-A induced lung lesions. Double staining for LPCAT1 (an AEC2 marker) and Tm4sf1 (Fig. 8A) revealed that LPCAT1 positive cells with high expression of Tm4sf1, which identifies Tm4sf1^high^AEC2 cells as AEP cells, were consistently observed in the lesions from immediately after exposure to the end of the 18-week recovery period (Fig. 8B). However, AEPs (Tm4sf1^high^AEC2) were not observed in normal lungs or bronchiolo-alveolar hyperplasia (Fig. 8B).

**Fig. 9:**
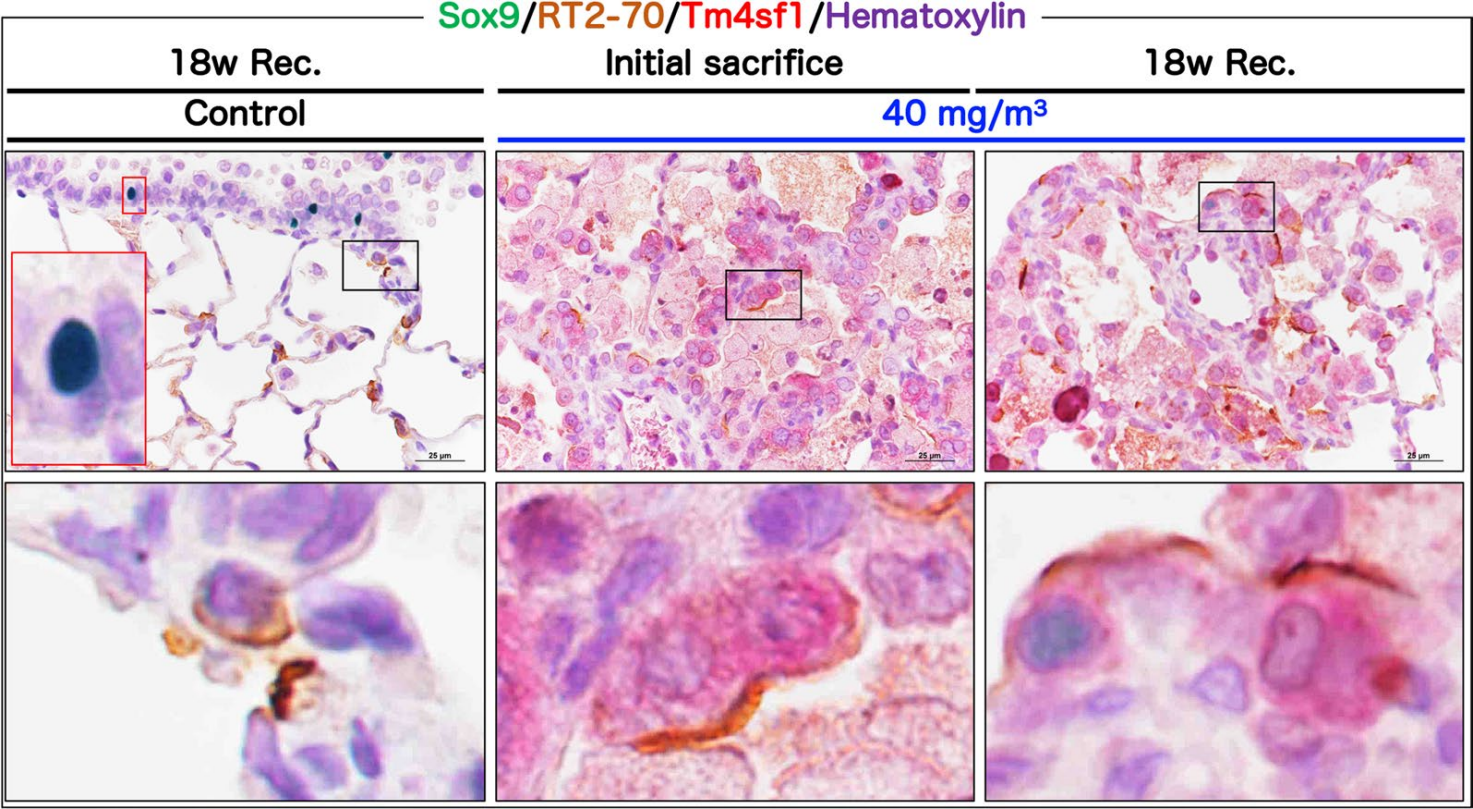
Triple staining for Sox9 (green in nucleus), RT2-70 (an AEC2 marker, brown in the cell membrane), and Tm4sf1 (an AEP marker, red in the cytoplasm) in the rat lung. All data are the results of the high-concentration intermittent inhalation experiment

Laughney et al. performed single-cell analysis using various lung tumor samples and revealed the existence of Sox9-positive AEP cells as a variant AEP [19]. We then examined whether Sox9-positive AEPs are present in CWAAP-A induced lung lesions. RT2-70 (also known as RTII70 and RTII-70) is reported to be an AEC2 specific marker [20, 21]. To investigate the usefulness of RT2-70 as a cell membrane marker of AEC2, sections were double-stained for RT2-70 and LPCAT1 or ATP binding cassette subfamily A member 3 (ABCA3), which are cytoplasmic markers of AEC2. RT2-70 was co-expressed in almost all LPCAT1-positive and ABCA3-positive cells in both normal and CWAAP-A exposed lung (Additional file 9: Fig. S9), thus confirming RT2-70-positive cells are AEC2s. The result of triple-staining for Sox9, Tm4sf1, and RT2-70 showed that Sox9-positive AEC2s and AEPs (Tm4sf1^high^AEC2) were not observed in normal lungs (Fig. 9 left panels), while a few Sox9-Tm4sf1-RT2-70 triple-positive cells were observed in 40 mg/m^3^ exposed lungs after the 18-week recovery period (Fig. 9 right panels).

These results are consistent with our previous report [13] and confirm that AEPs are conserved in the rat lung and may be involved in the formation and progression of CWAAP-A induced lung lesions. Furthermore, these results support the premise that AEP expansion in the lesion represents a regenerative change in the alveoli in response to alveolar toxicity caused by exposure to CWAAP-A.

### CWAAP-A causes alveolar injury in the acute phase

**Fig. 10:**
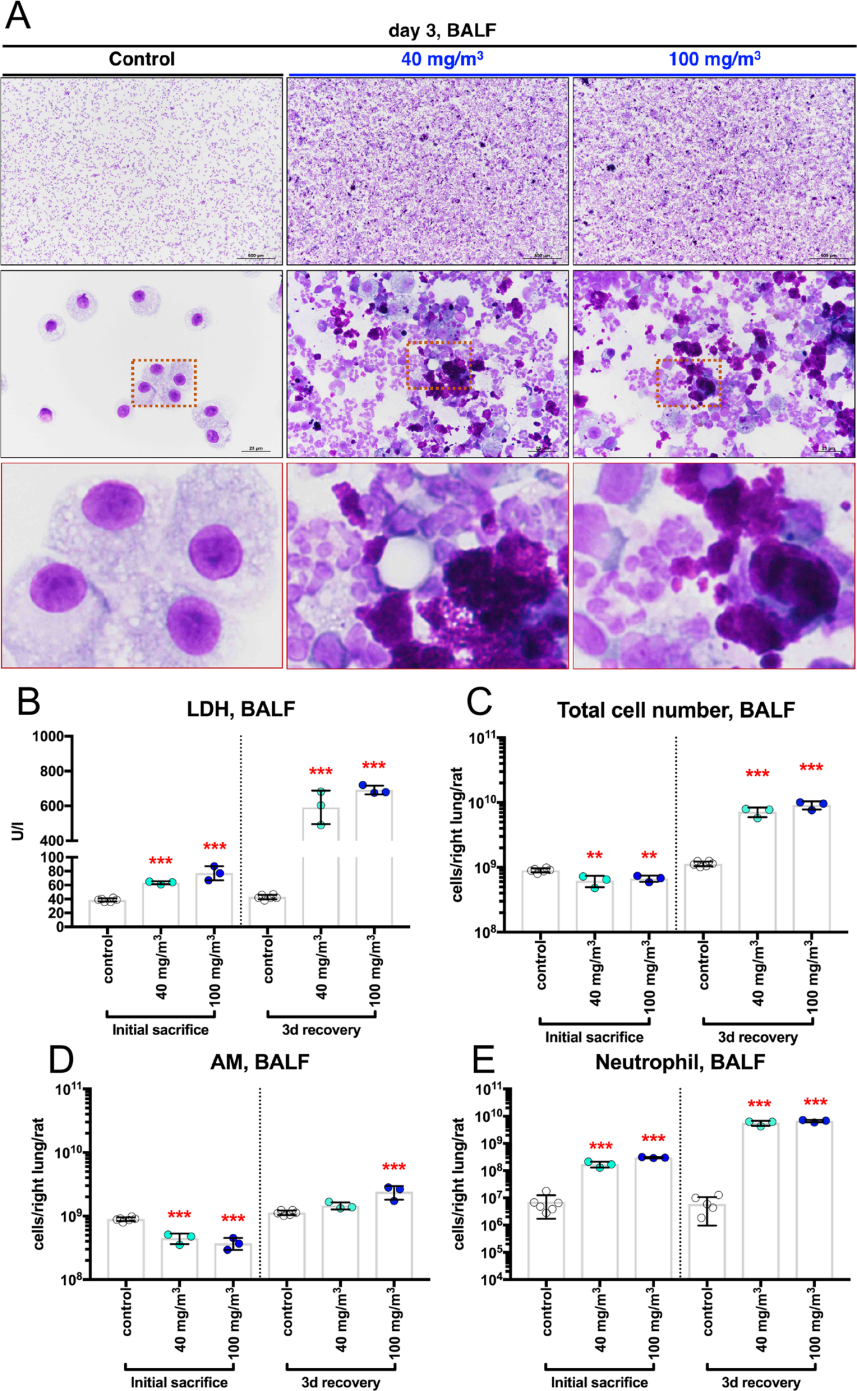
BALF collected from male rats after a single inhalation exposure to CWAAP-A (40 or 100 mg/m^3^). Representative images of the BALF cytospin cytology (**A**). Lactate dehydrogenase (LDH) activity (**B**), total cell number (**C**), alveolar macrophage number (**D**), and neutrophil number (**E**) in the BALF. Statistical significance was analyzed using Dunnett’s multiple comparison test: **p < 0.01 and ***p < 0.001 versus controls. *3d* day 3

**Fig. 11:**
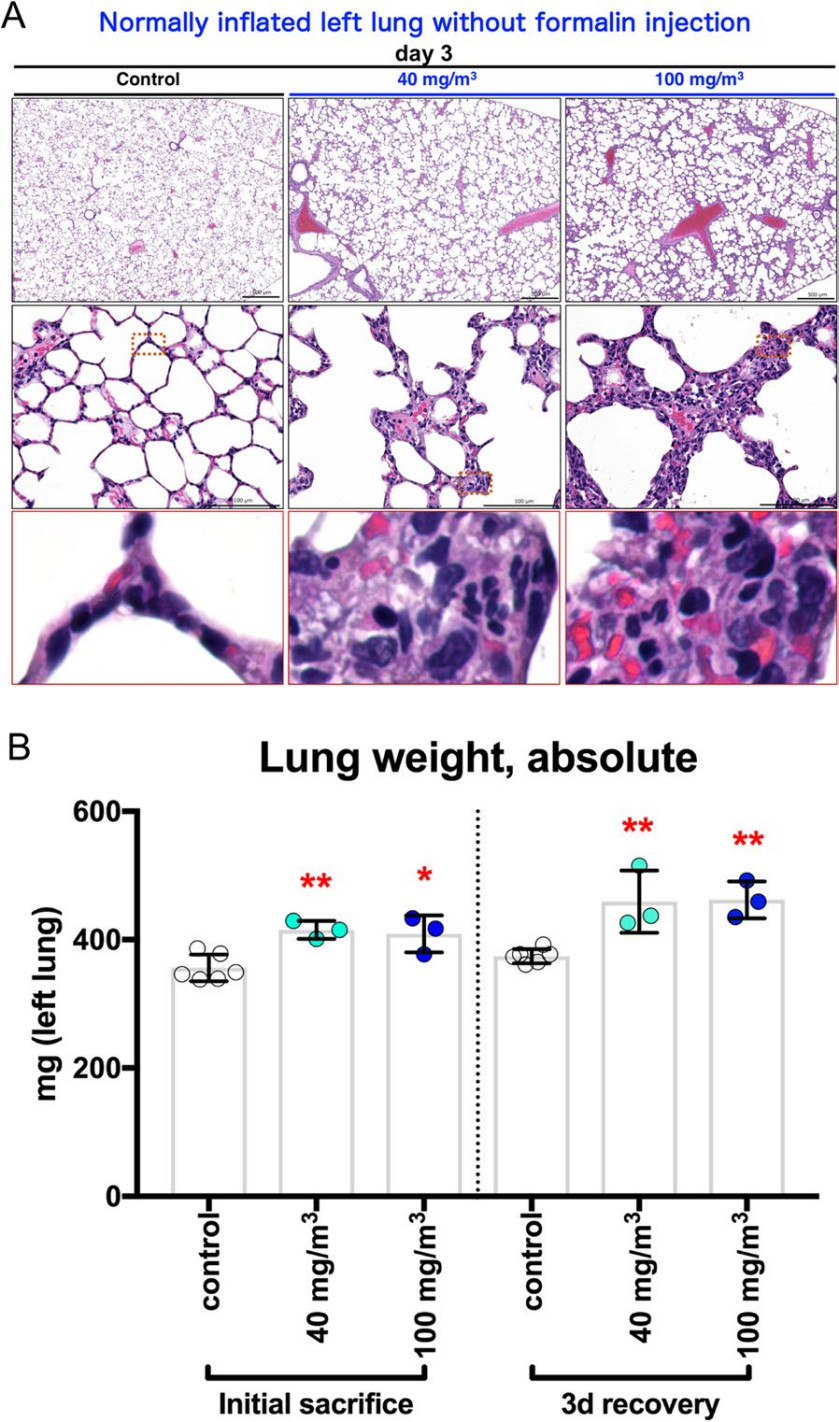
The effect of a single inhalation exposure to CWAAP-A on the pathology and weight of the lungs. Representative histopathological photographs of the left lung inflated by air (**A**) and left lung weight (**B**). Statistical significance was analyzed using Dunnett’s multiple comparison test: *p < 0.05 and **p < 0.01 versus controls

In order to clarify what happened in the early stage of alveolar lesion development induced by CWAAP-A, a single inhalation exposure test was conducted. In accordance with the repeated inhalation exposure study, a large number of neutrophils and CWAAP-A were found in the BALF three days after exposure (Fig. 10A). In addition, the LDH activity in the BALF showed a significant increase compared to the control group at 1 h and 3 days after exposure in both the 40 and 100 mg/m^3^ groups (Fig. 10B). The significant increase in the total number of cells in the BALF in the exposed groups three days after exposure was clearly due to neutrophils (Fig. 10C, E). These results indicate that a single exposure to CWAAP-A causes significant increases in LDH activity in the BALF and induces neutrophil infiltration into the lungs. To obtain more accurate pathological images of the lungs, we developed a method to inflate lungs with air only, without injecting formalin solution into the lungs which is routinely done (Additional file 10: Fig. S10). In this method, the trachea is clipped shut before opening the chest to prevent air from escaping from the lung exposed to atmospheric pressure (Additional file 10: Fig. S10). Histopathological images of the left lung inflated with air alone and the right lung inflated with formalin injection were compared, and good expansion of alveoli was confirmed in both cases (Additional file 10: Fig. S10). Histopathological evaluation of the left lung of the single-exposed animals using this method revealed a large number of neutrophils around collapsed alveoli in the alveolar region (Fig. 11A) accompanied by elevated inflation of the surrounding alveolar ducts (Fig. 11A center and right panels). These pathological findings are shown in Table 2. Alveolar collapse with inflammatory cell infiltration was specifically observed animals 3 days after exposure to 40 and 100 mg/m^3^ CWAAP-A. Interestingly, these findings were not observed in the pathological specimens of the right lung, which were inflated by formalin injection (Additional file 11: Fig. S11). In addition, a significant increase in lung weight was observed in both the 1-h and 3-day post-exposure autopsy groups (Fig. 11B). These results indicate that a single inhalation exposure to CWAAP-A causes alveolar injury characterized by alveolar collapse with a high degree of neutrophilic infiltration in the acute phase.

**Table 2:** Histopathological findings of the lung after a single inhalation exposure to CWAAP-A

Experimental period	Initial sacrifice			3 d recovery		
Exposure concentration (mg/m^3^)	0	40	100	0	40	100
No. of animals examined	6	3	3	6	3	3
Histopathological findings						
Lung						
Alveolar collapse with inflammatory cell infiltration	0	0	0	0	3**	3**
					< 2 >	< 2 >

Values indicate number of animals bearing lesions

The value in angle bracket indicate the average severity grade index of the lesion. The average severity grade is calculated using the following equation: Σ(grade × number of animals with grade)/number of affected animals

Grade: 1, slight; 2, moderate; 3, marked; 4, severe. Significant difference: *p < 0.05; **p < 0.01; ***p < 0.001 by Chi square test compared with the respective controls

### Intratracheal instillation is useful for the screening of CWAAP-induced rat pulmonary disorders

**Fig. 12:**
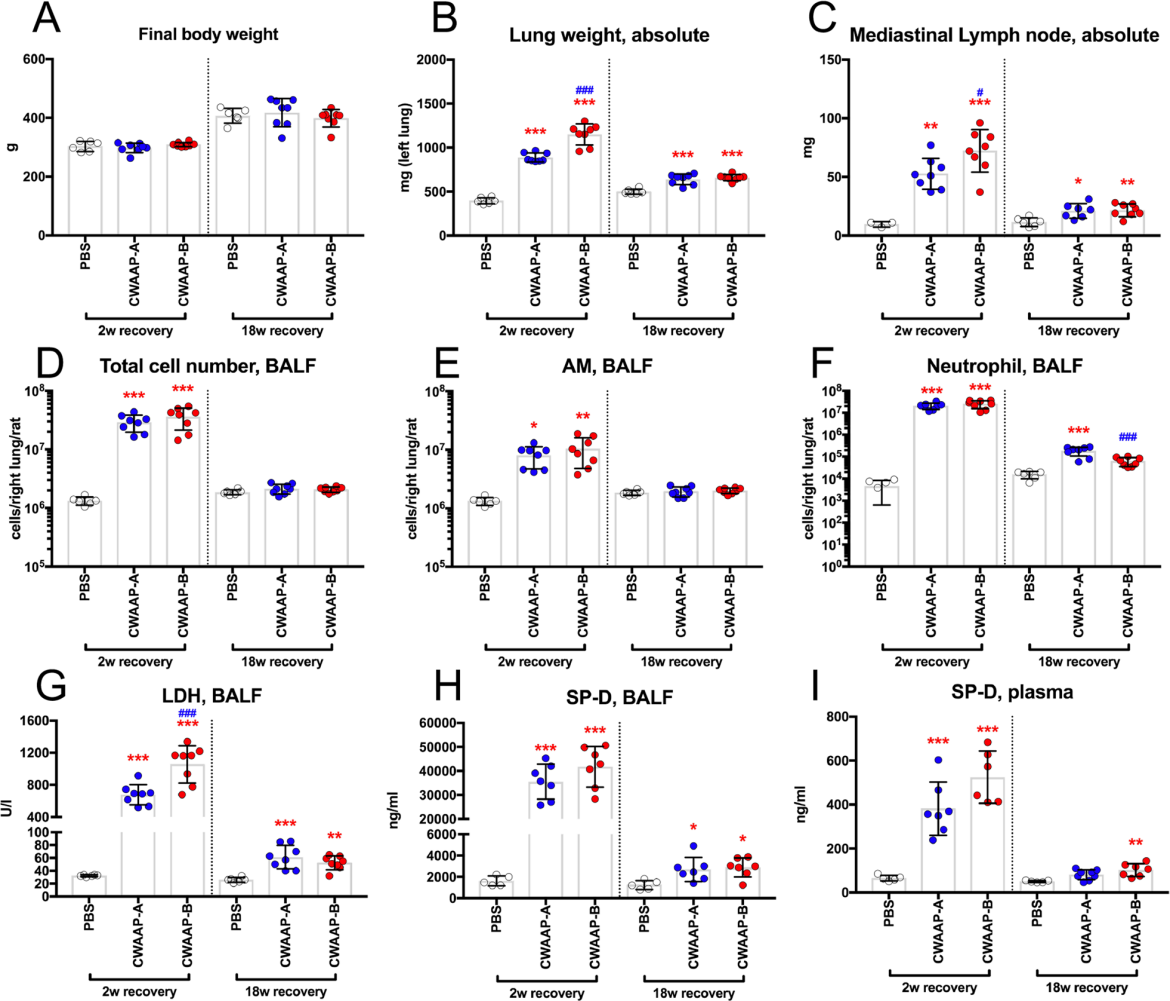
CWAAP-A or CWAAP-B (both administered at 1 mg/kg per dose) administered to male rats by repeated intratracheal instillations (see Additional file 2: Fig. S2C for details). Final body weights (**A**), left lung weights (**B**), mediastinal lymph node weights (**C**), total cell number in the BALF (**D**), alveolar macrophage number in the BALF (**E**), neutrophil number in the BALF (**F**), LDH activity in the BALF (**G**), SP-D level in the BALF (**H**), and SP-D level in the plasma (**I**) were measured at each sacrifice. Tukey’s multiple comparison tests were carried out for each sacrifice: *p < 0.05, **p < 0.01, and ***p < 0.001 indicate significant differences from the control (PBS) group, and ^#^*p* < 0.05 and ^###^*p* < 0.001 indicate significant differences between CWAAP-A and CWAAP-B

**Fig. 13:**
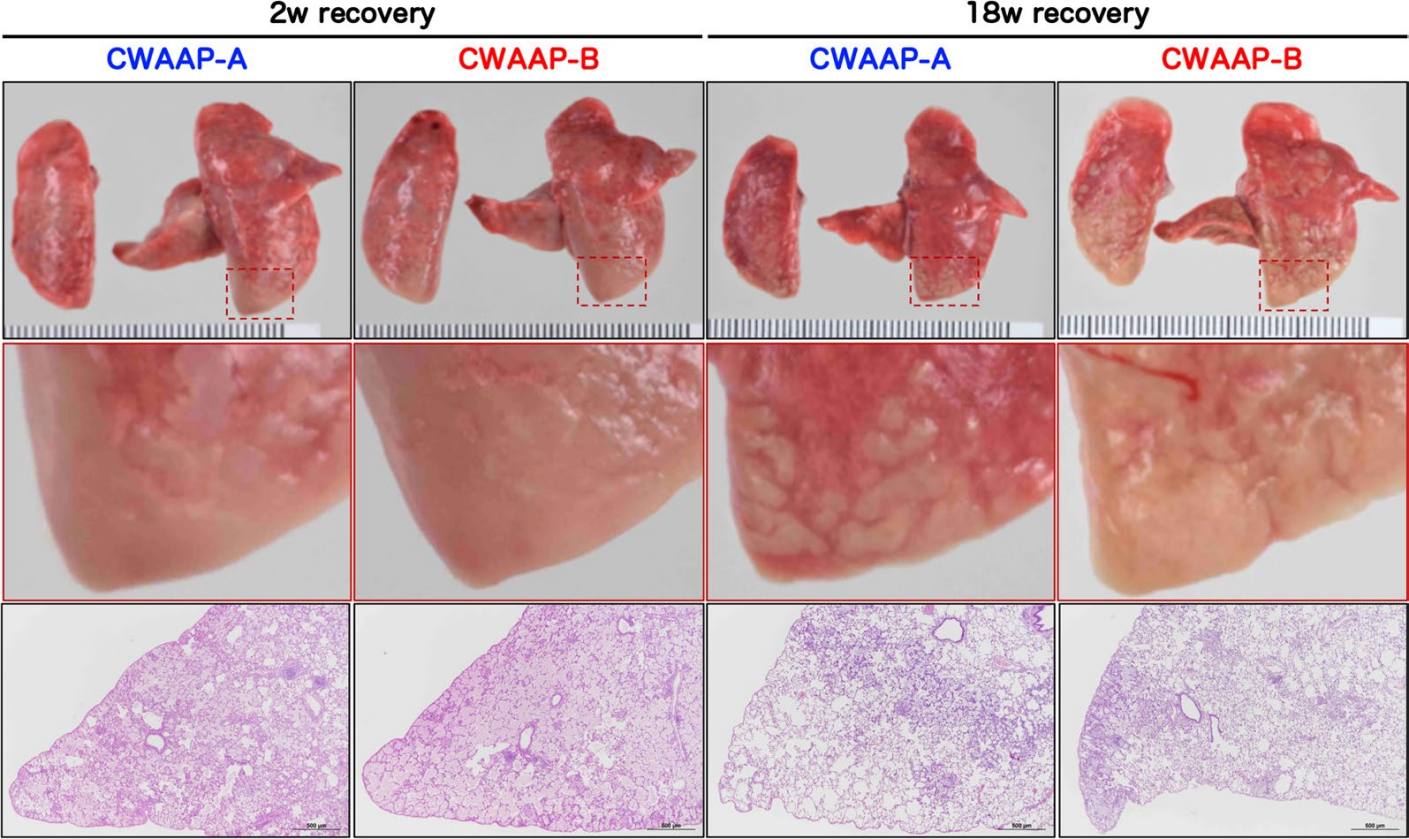
Representative macroscopic and microscopic photographs of rat lungs after intratracheal instillation of CWAAPs. White spots were observed on the caudal side of the right and left lungs (middle panels), and histopathological images of the same areas showed diffuse lesions (lower panels)

Although the intratracheal instillation method is useful for low-cost and simple toxicity assessment of a large number of chemicals, it is unclear whether this can be applied to CWAAPs, which are characterized by a type of thickener that improves viscosity and sol–gel stability. Therefore, we conducted an intratracheal instillation study using CWAAP-A, which was used in the whole-body inhalation exposure study, and another CWAAP, CWAAP-B. The administration and sampling schedule were similar to that of the inhalation exposure study. The final body weight was not affected by the intratracheal administration of either of the CWAAPs (Fig. 12A). In sharp contrast, lung weight (Fig. 12B) and mediastinal lymph node weight (Fig. 12C) were dramatically increased in both the CWAAP-A and CWAAP-B 2-week recovery groups. The increases in lung weight and mediastinal lymph node weight were less after the 18-week recovery period, but they were still significantly elevated. A similar trend was also observed for other lesion markers: total cell count in the BALF (Fig. 12D), alveolar macrophage count (Fig. 12E), neutrophil count (Fig. 12F), LDH activity (Fig. 12G), and SP-D level (Fig. 12H) in the BALF, and SP-D level in the plasma (Fig. 12I) were all markedly increased compared to controls 2 weeks after CWAAP administration and these increases had decreased or disappeared at 18 weeks after CWAAP administration. Representative lung and histopathological images are shown in Fig. 13. Because CWAAPs were administered intratracheally as a liquid suspension in phosphate-buffered saline (PBS), the foci were distributed caudally in the left and right lungs (Fig. 13). The histopathological findings of the lungs and mediastinal lymph nodes observed in this study are shown in Table 3 and are qualitatively similar to the results of the systemic inhalation exposure study (Table 1).

**Table 3:** Histopathological findings of the mediastinal lymph node and lung after repeated intratracheal instillation of CWAAPs

Experimental weeks	2w recovery			18w recovery		
Treatment	PBS	CWAAP-A	CWAAP-B	PBS	CWAAP-A	CWAAP-B
No. of animals examined	6	8	8	6	8	8
Histopathological findings						
Mediastinal lymph node						
Lymphoid hyperplasia	0	8***	8***	0	4*	6**
		< 2 >	< 2 >		< 1.3 >	< 1 >
Lung						
Deposition of test compound (AB-positive material)	0	8***	8***	0	0	0
		< 1 >	< 1 >			
Inflammation, perivascular	0	8***	8***	0	0	0
		< 1 >	< 1.4 >			
Granulomatous change, alveolar	0	8***	8***	0	8***	8***
		< 2 >	< 1.6 >		< 1 >	< 1 >
Hypertrophy/proliferation of alveolar epithelium	0	8***	8***	0	8***	8***
		< 2 >	< 2 >		< 1.4 >	< 1.3 >
Inflammation, air space	0	8***	8***	0	8***	8***
		< 2 >	< 2 >		< 1 >	< 1 >
Cholesterol cleft, air space	0	0	0	0	8***	8***
					< 1 >	< 1 >
Alveolitis	0	0	0	0	8***	8***
					< 2 >	< 1.9 >
Fibrous thickening, interstitial	0	0	0	0	8***	8***
					< 1 >	< 1 >
Accumulation of lipoproteinous material, air space	0	8***	8***	0	0	0
		< 2.6 >	< 3 >			
Hyperplasia, bronchiolo-alveolar	0	0	0	0	1	0
					< 1 >	

Values indicate number of animals bearing lesions

The value in angle bracket indicate the average severity grade index of the lesion. The average severity grade is calculated using the following equation: Σ(grade × number of animals with grade)/number of affected animals

Grade: 1, slight; 2, moderate; 3, marked; 4, severe. Significant difference: *p < 0.05; **p < 0.01; ***p < 0.001 by Chi square test compared with the respective controls

There were differences in the toxicity of CWAAP-A and CWAAP-B. Significant increases were observed in lung weight (Fig. 12B), mediastinal lymph node weight (Fig. 12C), and LDH level in the BALF (Fig. 12G) of rats administered CWAAP-B compared to rats administered CWAAP-A after the 2-week recovery period. Consistent with these results, the histopathological analysis showed that the accumulation of lipoproteinous material, an important indicator of alveolar proteinosis, tended to be greater in CWAAP-B exposed lungs than in CWAAP-A exposed lungs (Table 3). However, these differences disappeared after the 18-week recovery period. These results indicate that serial intratracheal administration can detect the pulmonary toxicity of CWAAPs in rats. Furthermore, it is useful for comparing the intensity of pulmonary damage caused by different CWAAPs. Our results suggest that CWAAP-B has a stronger damaging effect in the lung than CWAAP-A.

## Discussion

**Fig. 14:**
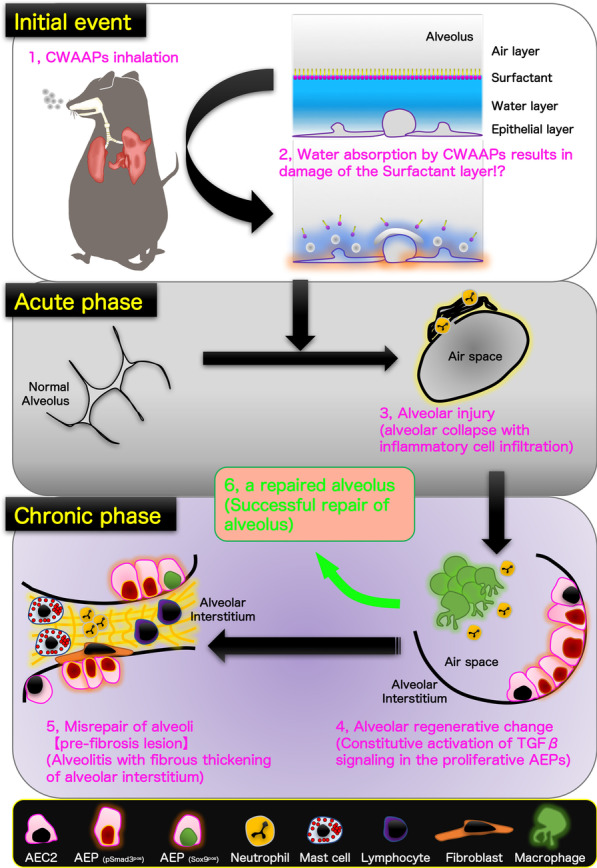
Graphical abstract for this study. A brief summary of the process by which alveolitis was caused by inhalation of CWAAPs is given below. (1) Inhalation of CWAAPs can damage (2) the surfactant layer by retaining water on the alveolar surface causing (3) alveolar collapse with inflammatory cell infiltration, mainly neutrophils, in the acute phase. Alveolar collapse can persist for up to 3 days after a single 4 h exposure to CWAAP-A. Alveolar collapse may recover after cessation of exposure, but persistent exposure can lead to (4) persistent inflammation and alveolar epithelial hypertrophy/proliferation. Immediately after repeated exposure to CWAAP-A, inflammation and alveolar epithelial hypertrophy/proliferation were observed throughout the lungs of the CWAAP-A exposed rats. These findings were accompanied by the appearance alveolar epithelial progenitor (AEP) cells, suggesting a regenerative change after lung injury. After an 18-week recovery period, inflammatory changes in the alveoli (4) partially led to a repaired alveolus (successful repair of alveolus) (6), as evidenced by a decrease in LDH in the BALF, but some developed into (5) alveolitis with fibrous thickening. This change is a "pre-fibrosis lesion" and is considered irreversible (regarded as misrepair of alveoli)

In this study, we examined the cellular and molecular mechanisms related to pulmonary disease in CWAAP-exposed F344 rats using a protocol that was relevant to the exposure environment of workers that developed interstitial lung diseases at a CWAAP manufacturing site. The results showed that both high-concentration intermittent (repeated) exposures and intratracheal instillation (as an adjunct to inhalation exposure) produced lung pathologies similar to those produced in our previous CWAAP-A inhalation study [8]. In addition to this, the present study also included an experiment in which rats were exposed once to high-concentrations of CWAAP-A. The results of the single-exposure experiment and the repeated-exposure experiment demonstrated that inhalation exposure to CWAAP-A caused alveolar collapse and neutrophil infiltration into the lung and this progressed to fibrosis. At the cellular level, TGFβ signaling in alveolar epithelial type 2 cells (AEC2) and alveolar epithelial progenitor cells (AEPs) and expansion of AEPs were primary events in the progression of CWAAP-A induced pulmonary lesions to pulmonary fibrosis. A brief summary of the process by which alveolitis was caused by inhalation of CWAAPs in rats revealed in this study is shown in Fig. 14.

To compare the toxicological events between experiments with different exposure protocols of the same test compound the cumulative delivered doses (multiplying the concentration in the test atmosphere by the total number of hours of exposure: expressed as mg/m^3^ × h) should be determined [22]. The cumulative doses for the present study and our previous 13-week subchronic study [8] are shown in Table 4. The cumulative dose for the 40 mg/m^3^ exposure level in the present study was 1440 mg/m^3^, and the cumulative dose for the for 10 mg/m^3^ exposure level (the highest exposure level) in the previous study was 3900 mg/m^3^. Despite this approximately 2.7-fold difference in overall exposure level, these two groups had similar findings and severity in histopathological parameters, especially lung lesions (Table 5) and LDH levels in the BALF (Table 4), with both groups showing almost the same fold change with respect to their respective control groups. These results suggest that short-duration exposure of test animals to high-levels of CWAAP-A is a valid protocol and may be more relevant than long-duration exposure of test animals to middle-to-low concentrations of CWAAP-A for investigating diseases observed at actual occupational accident sites.

**Table 4:** Comparison of the effect of inhalation exposure to CWAAP-A in this study and our previous 13-week study (reference: Takeda et al. [8])

Exposure protocol	Chamber condition		Cumulative dose	LDH in BALF	
Hours/day, days/week, total hour	MMAD (GSD) (μm)	Exposure concentration (mg/m^3^)	Dose (mg/m^3^ × h)	Average ± SD (U/L)	Fold change
CWAAP-A (the present study, high-concentration intermittent exposure)					
Male F344/DuCrlCrlj rat					
4 h/day, 1 day/week for 2 months (total 36 h)		0	–	25 ± 1.1	–
	0.8 (2.4)	15	540	330 ± 11.7	13.0
	0.7 (2.4)	40	1440	636 ± 129.8	25.0
CWAAP-A (Our previous report: Reference: Takeda et al. [8])					
Male F344/DuCrlCrlj rat		0	–	25 ± 1.2	–
	0.8 (2.7)	0.3	117	62 ± 6.3	2.5
	0.8 (2.6)	1	390	177 ± 13.6	7.1
	0.8 (2.6)	3	1170	319 ± 17.6	12.8
	0.8 (2.7)	10	3900	684 ± 46.9	27.4
Female F344/DuCrlCrlj rat		0	–	29 ± 1.0	–
6 h/day, 5 days/week for 13 weeks (total 390 h)	0.8 (2.7)	0.3	117	60 ± 4.1	2.0
	0.8 (2.6)	1	390	140 ± 16.6	4.8
	0.8 (2.6)	3	1170	268 ± 10.4	9.2
	0.8 (2.7)	10	3900	681 ± 83.0	23.3

The data on LDH in BALF were taken from Fig. 6E of the present study and Fig. 11A, B of Reference: Takeda et al. [8]

**Table 5:** Comparison of the effects of inhalation exposure to CWAAP-A on histopathological findings in the lungs of male rats in the present study and in our previous 13-week study (Reference: Takeda et al. [8])

Experimental protocol	The present study			Our previous report: Takeda et al. 2022		
Exposure concentration (mg/m^3^)	40	40	40	10	10	10
Recovery period (weeks)	0	2	18	0	4	13
No. of animals examined	6	6	10	8	8	8
Histopathological findings						
Male lung						
Granulomatous change, alveolar	6	6	10	8	8	8
	< 2 >	< 2 >	< 1 >	< 1.9 >	< 1.9 >	< 1 >
Multifocal lesion, alveolar						
Hypertrophy/proliferation of alveolar epithelium	6	6	10	8	8	8
	< 3 >	< 2 >	< 2 >	< 2 >	< 2 >	< 1.8 >
Inflammation, air space	6	6	10	8	8	8
	< 3 >	< 2 >	< 1 >	< 2 >	< 2 >	< 1 >
Cholesterol cleft, air space	0	0	10	0	8	8
			< 1 >		< 1 >	< 1 >
Alveolitis	0	0	10	0	3	8
			< 2 >		< 1 >	< 2 >
Fibrous thickening, interstitial	0	0	10	0	3	8
			< 1 >		< 1 >	< 1 >
Accumulation of lipoproteinous material, air space	6	6	0	8	6	8
	< 2.2 >	< 1.2 >		< 1.9 >	< 1 >	< 1 >

Values indicate number of animals bearing lesions

The values in angle brackets indicate the average severity grade index of the lesion. The average severity grade is calculated using the following equation: Σ(grade × number of animals with grade)/number of affected animals

Grade: 1, slight; 2, moderate; 3, marked; 4, severe

These data are shown in Table 1 in the present study and Tables 2, 3, and 4 in Reference Takeda et al. [8]

In this study, we used a single inhalation exposure to CWAAP-A to investigate the lung damage of CWAAP-A in the acute phase, and utilized an alternative sampling method (Additional file 10: Fig. S10) that reproduces as closely as possible the inflation of rat lungs without formalin inflation. We found that in the rat lung in the acute phase after CWAAP-A inhalation, alveolar collapse with a high degree of neutrophil infiltration occurred (Fig. 14, middle panel). If the lung remains collapsed and the resting tissue is filled with edema, gas exchange will be defective and pneumonia will likely occur [23, 24]. If the alveoli are not reopened, the pathologic lung will progress to fibrosis [25–27]. Therefore, the CWAAP-A induced alveolar collapse in the acute phase may trigger pneumonia and the fibrotic changes observed in the chronic phase.

Typical diseases that cause alveolar injury are acute respiratory distress syndrome (ARDS) and ventilator-induced lung injury secondary to mechanical ventilation, which is the only treatment for ARDS [28]. ARDS is a complex syndrome characterized by four well-accepted central components [29] known as the pathologic tetrad [30]. When loss of surfactant function (surfactant deactivation) [31] occurs, the surface tension of the alveoli increases, which is known to exacerbate the increase in edema fluid in the alveoli [32]. Surfactant dysfunction alters alveolar mechanics, resulting in recruitment/derecruitment of alveoli with each breath [33]. Surfactant deactivation exacerbates alveolar collapse over time by promoting instability and collapse of the heterogeneous lung tissue. Consistent with these components, the present study found that a single exposure to CWAAP-A resulted in alveolar collapse with neutrophilic infiltration and elevated inflation of alveolar ducts around collapsed alveoli in rat lungs 3 days after exposure. However, hyaline membrane formation, hemorrhage, and plasma pulmonary edema were not observed. These results suggest that CWAAP-A exposure induces alveolar collapse in rat lungs, but that direct damage to epithelial cells and endothelial cells is minor. It is commonly understood that CWAAPs are dispersed in water at the molecular level, and unlike super absorbent polymers swell by water incorporated into their cross-linked molecular structure [34–36]. Thus, water intake by inhaled CWAAP occurs on the alveolar epithelial surface (the water layer) and damages the surfactant layer, thereby altering alveolar mechanics and causing alveolar collapse and inflammation (upper panel of Fig. 14). Thus, in contrast to ARDS, which is caused by increased vascular permeability and severe epithelial/endothelial injury, the acute phase of CWAAP-induced lung lesions in rats may be due to alveolar damage secondary to the disruption of the alveolar surfactant microenvironment that occurs after CWAAP disrupts the water layer.

The present study found that alveolitis with fibrous thickening of the alveolar septa occurs as a typical chronic lesion caused by CWAAP-A. Although most of the inflammatory lesions were restored to normal after an 18-week recovery period following the final exposure to CWAAP-A (Green arrow from alveolar regenerative change to a repaired alveolus, Fig. 14), some of these lesions progressed as a pathological condition. Alveolitis is the basic pathological term for interstitial pneumonia. Idiopathic pulmonary fibrosis, a classic example of interstitial pneumonia with fibrosis, is one of the most studied areas of respiratory diseases [37–39], and TGFβ signaling plays a central role in the pathogenesis of pulmonary fibrotic disease [9, 40]. TGFβ signaling is activated by the binding of ligands, including TGFβ1 and TGFβ2, to their receptors on the cell surface, and regulates downstream transcriptional networks through phosphorylation and nuclear translocation of Smad2 and Smad3 [10]. Phosphorylation of serine residues (S423 and S425) at the C-terminus of the MH2 domain of the Smad3 protein is essential for activating the TGFβ signaling transcriptional network [41]. In the present study, using an antibody that recognizes phosphorylated serine residues of Smad3, we found that persistent TGFβ signaling was activated in AEC2s in multifocal lesions even after an 18-week recovery period following CWAAP-A exposure. A previous study using a mouse model of lipopolysaccharide-induced lung injury reported that activation of TGFβ signaling in AEC2s is required for cell cycle arrest but inhibits differentiation to AEC1 [42]. Further studies focusing on the molecular mechanisms by which TGFβ signaling in proliferating AEC2s continues to be activated are needed to better understand the pulmonary toxicity of CWAAPs.

CWAAP-A induced alveolar lesions consist of enlarged and proliferating AEC2s for alveolar regeneration. Recently, single-cell analysis has become widely used to identify the cell populations that compose normal and diseased lungs, especially disease-specific cell populations [43–45]. It has been reported that AEPs are present in mouse and human lungs to contribute to the activation of regenerative molecular programs [11, 12] and that Sox9-positive AEPs also exist [19]. Although in this study, we were not able to identify AEPs in normal rat lungs, consistent with our previous findings in rats [13], AEPs were observed immediately after CWAAP-A exposure and after the 18-week recovery period, suggesting that CWAAP-A induced lung lesions are regenerative changes, consistent with previous reports [11]. Furthermore, a small number of Sox9-positive AEPs were also observed in the lesions after the 18-week recovery period. This study is the first report to discuss the relevance of the presence of AEPs in CWAAP-induced rat lung lesions.

This study did not identify the mechanism of alveolitis with fibrous thickening of the alveolar interstitium induced by CWAAP-A. It is well known that fibroblasts transdifferentiated from AEC2s via epithelial-mesenchymal transition (EMT) are candidates for collagen-producing cells in pulmonary fibrosis [40, 46]. TGFβ signaling is also an important signal for the induction of EMT [9]. Recently, Li et al. reported that the TGFβ-Sox9 axis produces collagen 10a1 in EMT-mediated gastric cancer cells, and demonstrated that Sox9 binds directly to the promoter region of the col10a1 gene [47]. We found a high ratio of both phospho-Smad3-positive AEC2s and AEPs within CWAAP-A induced lung lesions. Since CWAAP-A induced lung lesions transitioned to alveolar interstitial-predominant lesions in the chronic phase, an EMT-mediated trans-differentiation of AEPs into fibroblast and involvement of reprograming via Sox9 expression may have occurred. These possibilities need to be studied in the future.

The present study is the first to investigate the pulmonary toxicity of CWAAP-A in rats by comparison of both high-concentration intermittent inhalation exposure and intratracheal instillation. The results showed that the pathological lesions caused by intratracheally administered CWAAP-A were qualitatively similar to those caused by inhalation exposure, and changes in plasma and BALF biochemical and cytological parameters were also similar after both types of exposure protocols. These findings indicate that intratracheal instillation is useful for the initial assessment of acute and chronic toxicity of CWAAP-A. Furthermore, using intratracheal instillation a second CWAAP, CWAAP-B, was evaluated. Two weeks after the final administration of CWAAPs there was a significant increase in lung weight and higher LDH activity in the BALF of the rats administered CWAAP-B compared to CWAAP-A, suggesting that CWAAP-B may have higher pulmonary toxicity than CWAAP-A. Thus, intratracheal instillation, which is rapid, simple, and inexpensive, is effective for the initial investigation of pulmonary toxicity of different CWAAPs.

The present study has a limitation. While we succeeded in clarifying the pulmonary toxicity of CWAAP-A in rats in the acute phase up to the chronic phase, since animals were evaluated only up to 26 weeks we could not clarify the end-stage pathogenesis of CWAAP-treated lungs, such as whether alveolitis with fibrous thickening progresses to pulmonary fibrosis. This may be the reason that in this study there was no evidence of progressive fibrosis with a high degree of destruction and remodeling of alveolar structures. Notably, one case of bronchiolo-alveolar hyperplasia, a pre-neoplastic lesion, was observed in one CWAAP-A-exposed rat in the inhalation study and one CWAAP-A treated rat in the intratracheal instillation study. To evaluate any lung carcinogenicity associated with inflammatory-fibrotic diseases caused by CWAAP, longer-term studies are needed.

## Conclusions

In this study, we investigated the cellular and molecular mechanisms related to rat pulmonary toxicity after exposure to CWAAP-A, which has caused occupational lung diseases in Japanese workplaces in recent years. The results showed that a single exposure to a high level of CWAAP-A caused alveolar injury in the acute phase and that high-concentration intermittent (repeated) exposures caused regenerative changes in the alveoli. After the end of exposure, during the recovery period, the alveolar lesions partially recovered to normal, but the some progressed to alveolitis with fibrous thickening. TGFβ signaling in AEC2 and AEP cells and AEP cell expansion were primary events in the pathogenesis of CWAAP-A induced disease. Moreover, we compared the lung pathology of CWAAP-A administered by systemic inhalation exposure with that of CWAAPs administered by intratracheal instillation and found that the lung pathologies were similar. The use of intratracheal instillation as an adjunct to inhalation exposure is expected to greatly accelerate testing of respirable CWAAP products, and consequently, to significantly increase our understanding of the pulmonary toxicity of CWAAP products.

## Supplementary Information

**Additional file 1: Figure S1**. Representative images of CWAAP-A, CWAAP-B and the chemical structural formula of CWAAP. Representative images of CWAAP-A (A), CWAAP-B (B) and the chemical structural formula of CWAAP (C-E). Acrylic acid polymer is a polymerized product of acrylic acid with carboxyl groups (C) and is anionic because of a large amount of carboxyl groups in the molecule. Cross-linked acrylic acid polymers (CWAAPs) have the characteristics of absorbing and retaining a large amount of water (E), as the polymer chain expands when it contains moisture compared to its dry state (D).

**Additional file 2: Figure S2**. The three animal experimental protocols used in this study. The high-concentration intermittent (repeated) inhalation exposure study (A), the high-concentration single inhalation study (B), and the repeated intratracheal instillation study (C).

**Additional file 3: Figure S3**. The whole-body inhalation exposure system using in this study. The direct-injection whole body inhalation system (A). Exposure concentrations of CWAAP-A in each chamber (B). Representative scanning electron microscope (SEM) images of the CWAAP-A particles in the chambers (C). Cumulative frequency distribution graphs with logarithmic probability (D). The mass median aerodynamic diameter (MMAD) and geometric standard deviation (σg) in the chambers measured during the second and eighth exposures (E). Scale bar: 20 μm (panel C).

**Additional file 4: Figure S4**. Experimental protocol of a pilot study. Experimental protocol of a pilot study using a single intratracheal instillation of 0.5 or 1.5 mg/kg CWAAP-A and CWAAP-B (A). Total cell number (B) and LDH activity (C) in the BALF of CWAAPs-treated rats and their respective controls (PBS).

**Additional file 5: Figure S5.** Representative images of the alcian blue staining of the normal rat lung and slides. Representative images of the alcian blue staining of the normal rat lung are shown in A. In B, CWAAP-A and CWAAP-B were placed on the slides and directly stained using Alcian blue.

**Additional file 6: Figure S6.** Representative microscopic photographs of a normal rat lung (sham air) after the 18 week recovery period. All data are the results of high-concentration intermittent inhalation study.

**Additional file 7: Figure S7**. Representative histopathological photographs of the bronchus and bronchiole in the rat lung after repeated inhalation exposure to CWAAP-A (40 mg/m^3^). All data are the results of high-concentration intermittent inhalation study.

**Additional file 8: Figure S8**. Representative images of Tm4sf1 expression in the rat lung after repeated inhalation exposure to 40 mg/m^3^ CWAAP-A. All data are the results of high-concentration intermittent inhalation study.

**Additional file 9: Figure S9**. Figure S9. Representative images of the AEC2 markers co-staining. Representative images of the AEC2 membranous marker RT2-70 co-staining with AEC2 cytoplasmic markers LPCAT1 (A)and ABCA3 (B) in the rat lung.

**Additional file 10: Figure S10**. Sampling procedure of a single inhalation exposure study. Sampling procedure of the air inflated left lung without formalin injection (A) and comparative images of the right lung inflated by formalin and the left lung inflated by air (B).

**Additional file 11: Figure S11**. Representative lung histopathological images of a single inhalation exposure study. Representative histopathological images of the right lung inflated by formalin and the left lung inflated by air of a rat 3 days after exposure to 100 mg/m^3^: the right lung and the left lung are from the same animal.

**Additional file 12: Table S1**. List of primary antibodies used in this study.

**Additional file 13: Table S2**. Summary of differences between hotspot and white spot.

## Data Availability

The datasets used and analyzed during the current study are available from the corresponding authors on reasonable request.

## Abbreviations

ABCA3: ATP binding cassette subfamily A member 3
AEC1: Alveolar epithelial type 1 cell
AEC2: Alveolar epithelial type 2 cell
AEP: Alveolar epithelial progenitor
ARDS: Acute respiratory distress syndrome
BAHyp: Bronchiolo-alveolar hyperplasia
BALF: Bronchoalveolar lavage fluid
CWAAP: Cross-linked water-soluble acrylic acid polymer
DAPI: 4′,6-Diamino-2-phenylindole
EMT: Epithelial–mesenchymal transition
HE: Hematoxylin and eosin
LDH: Lactate dehydrogenase
LPCAT1: Lysophosphatidylcholine acyltransferase 1
MMAD: Mass median aerodynamic diameter
NBF: Neutral buffered formalin
PAS: Periodic acid Schiff
RT: Room temperature
SEM: Scanning electron microscope
σg: Geometric standard deviation
Smad3: Mothers against decapentaplegic homolog 3
Sox9: SRY-Box transcription factor 9
SP-D: Surfactant protein D
SUR: Lesion-surrounding tissues
TGF: Transforming growth factor
Tm4sf1: Transmembrane 4 L six family member 1
