# Supplementary figures and images for: Mechanisms of pulmonary disease in F344 rats after workplace-relevant inhalation exposure to cross-linked water-soluble acrylic acid polymers
Source: Respir Res. 2023 Feb 13;24:47. doi: 10.1186/s12931-023-02355-z (PMC9926550; doi:10.1186/s12931-023-02355-z)

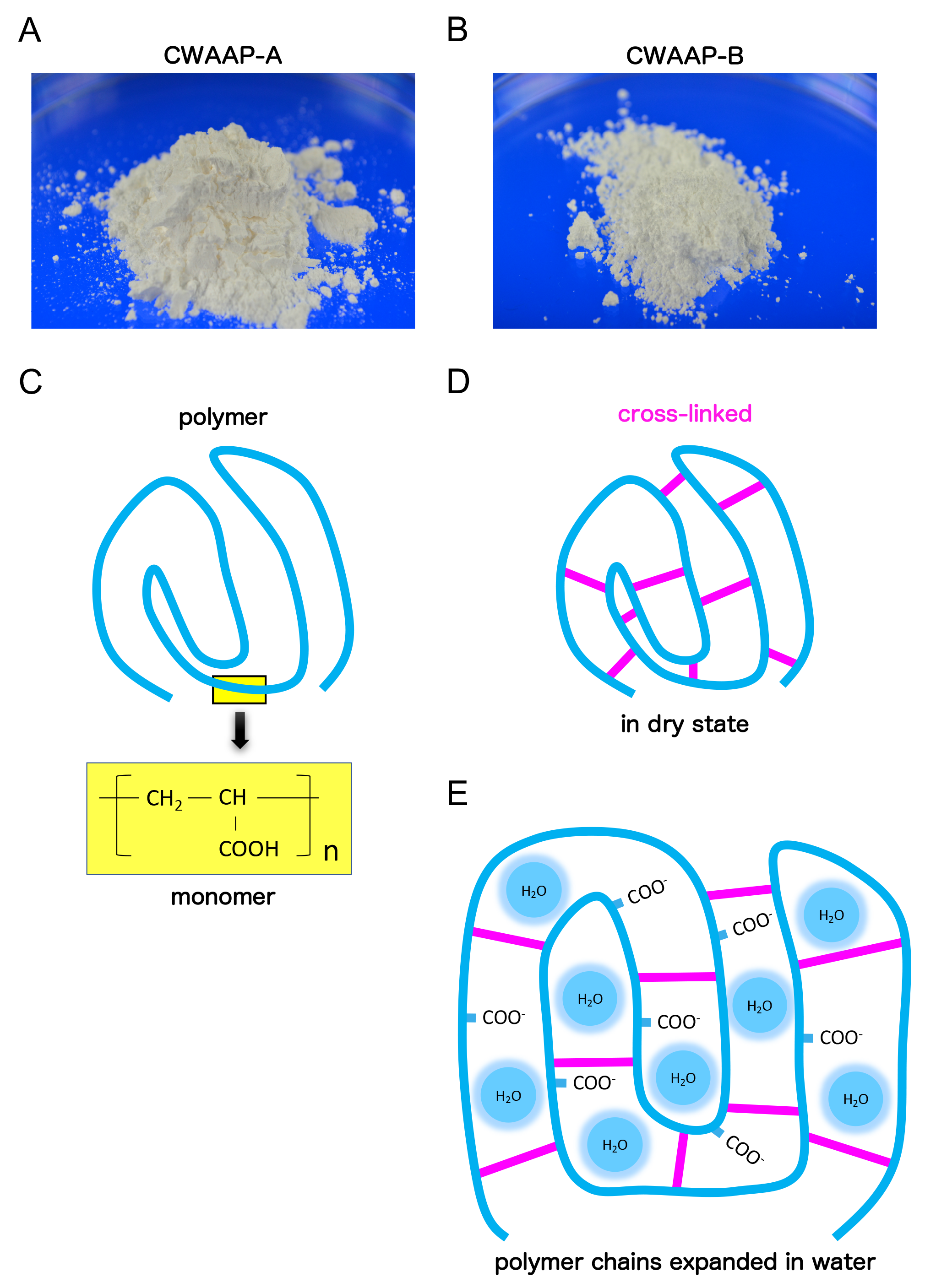

Supplement: Supplementary file 1 — Additional file 1: Figure S1. Representative images of CWAAP-A, CWAAP-B and the chemical structural formula of CWAAP. Representative images of CWAAP-A (A), CWAAP-B (B) and the chemical structural formula of CWAAP (C-E). Acrylic acid polymer is a polymerized product of acrylic acid with carboxyl groups (C) and is anionic because of a large amount of carboxyl groups in the molecule. Cross-linked acrylic acid polymers (CWAAPs) have the characteristics of absorbing and retaining a large amount of water (E), as the polymer chain expands when it contains moisture compared to its dry state (D). [file 12931_2023_2355_MOESM1_ESM.tif]

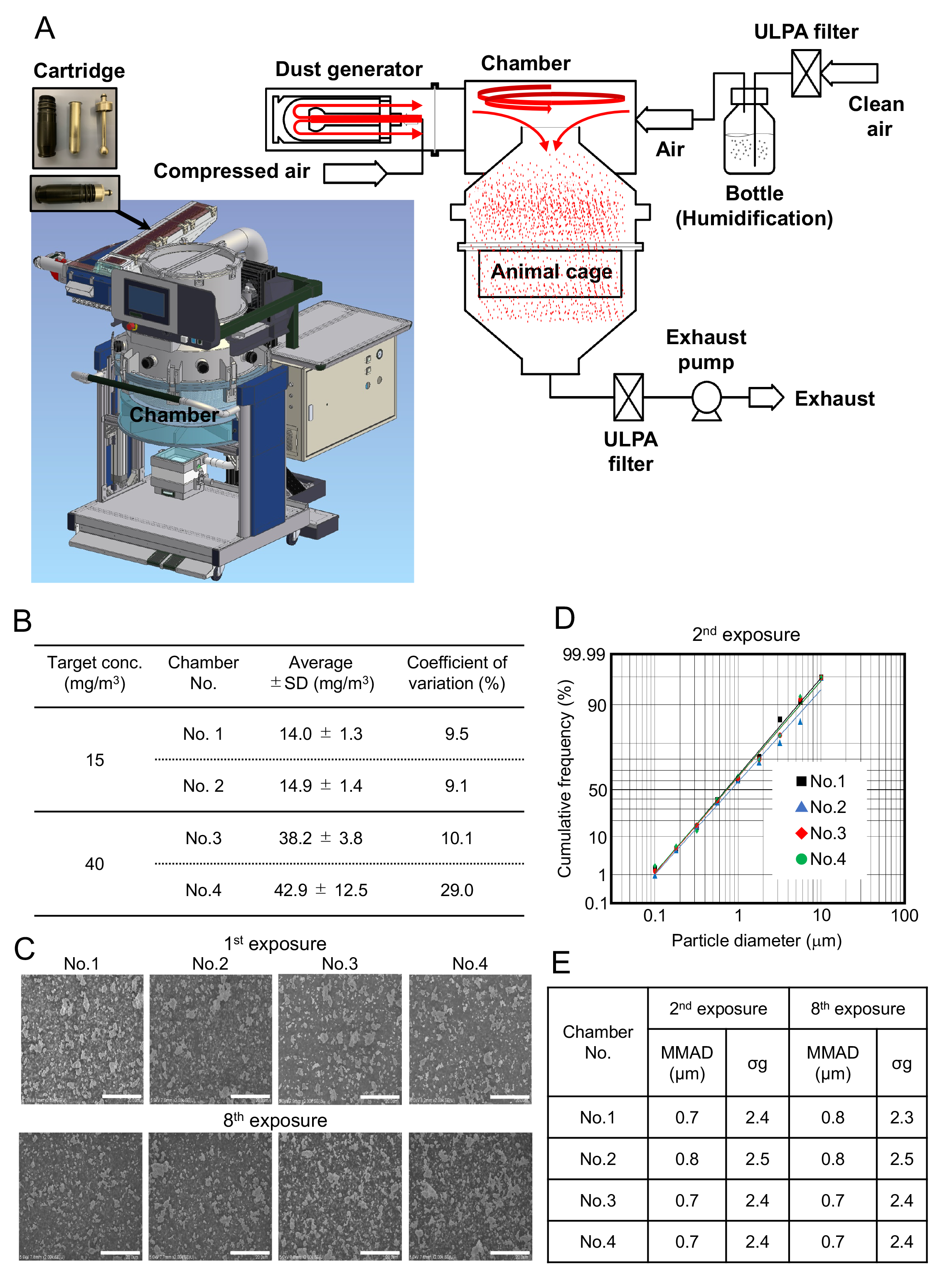

Supplement: Supplementary file 3 — Additional file 3: Figure S3. The whole-body inhalation exposure system using in this study. The direct-injection whole body inhalation system (A). Exposure concentrations of CWAAP-A in each chamber (B). Representative scanning electron microscope (SEM) images of the CWAAP-A particles in the chambers (C). Cumulative frequency distribution graphs with logarithmic probability (D). The mass median aerodynamic diameter (MMAD) and geometric standard deviation (σg) in the chambers measured during the second and eighth exposures (E). Scale bar: 20 μm (panel C). [file 12931_2023_2355_MOESM3_ESM.tif]

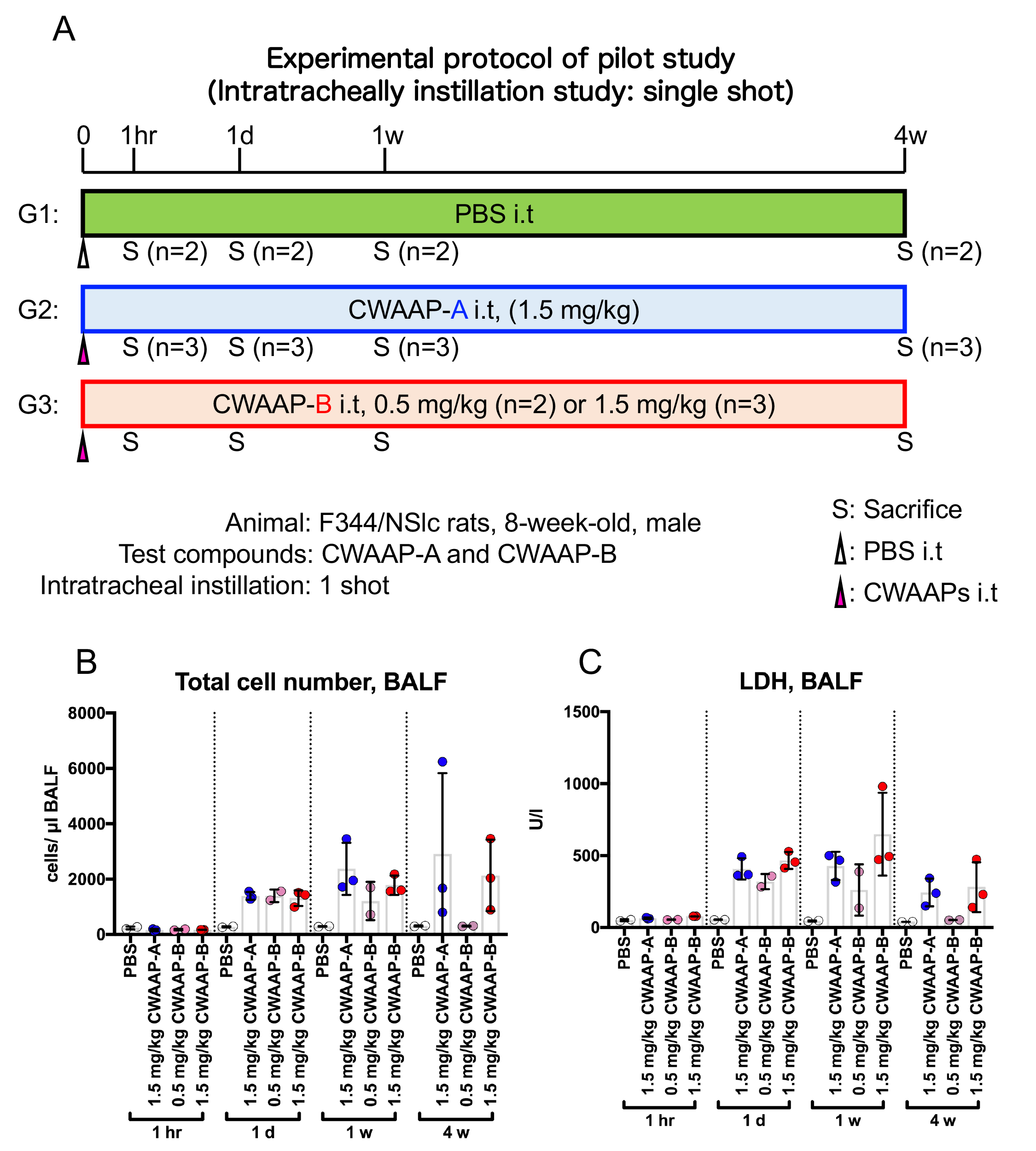

Supplement: Supplementary file 4 — Additional file 4: Figure S4. Experimental protocol of a pilot study. Experimental protocol of a pilot study using a single intratracheal instillation of 0.5 or 1.5 mg/kg CWAAP-A and CWAAP-B (A). Total cell number (B) and LDH activity (C) in the BALF of CWAAPs-treated rats and their respective controls (PBS). [file 12931_2023_2355_MOESM4_ESM.tif]

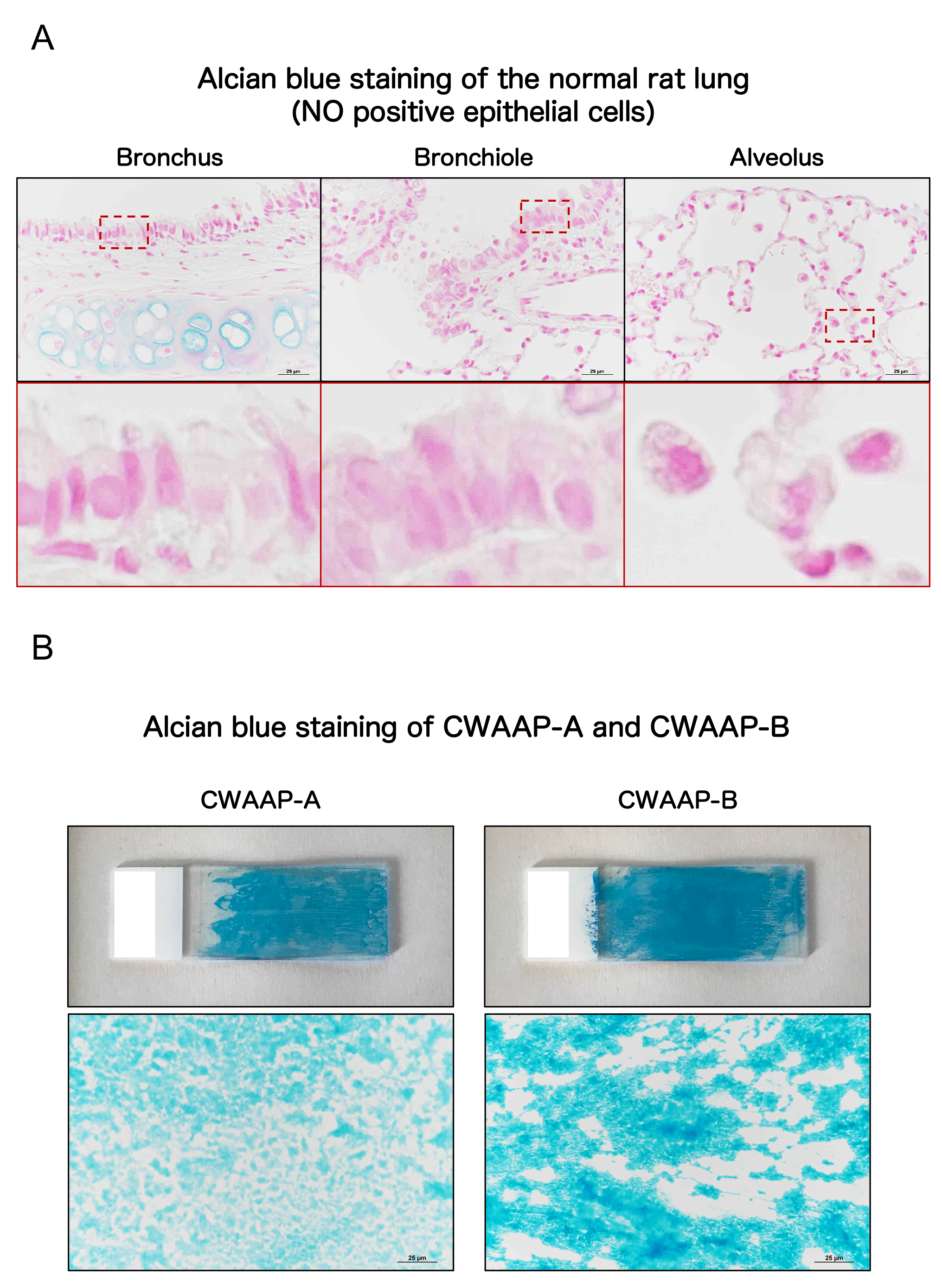

Supplement: Supplementary file 5 — Additional file 5: Figure S5. Representative images of the alcian blue staining of the normal rat lung and slides. Representative images of the alcian blue staining of the normal rat lung are shown in A. In B, CWAAP-A and CWAAP-B were placed on the slides and directly stained using Alcian blue. [file 12931_2023_2355_MOESM5_ESM.tif]

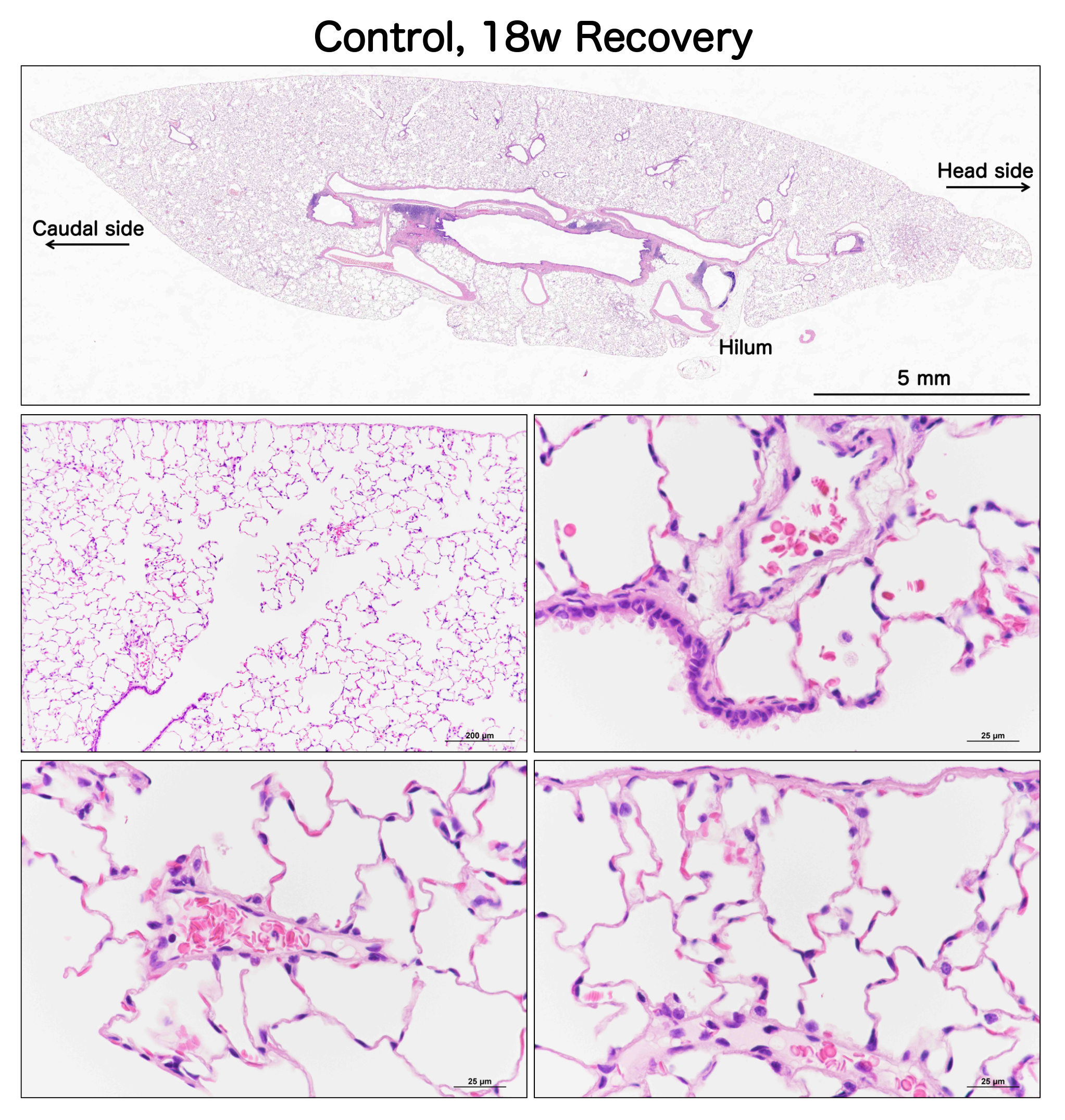

Supplement: Supplementary file 6 — Additional file 6: Figure S6. Representative microscopic photographs of a normal rat lung (sham air) after the 18 week recovery period. All data are the results of high-concentration intermittent inhalation study. [file 12931_2023_2355_MOESM6_ESM.tif]

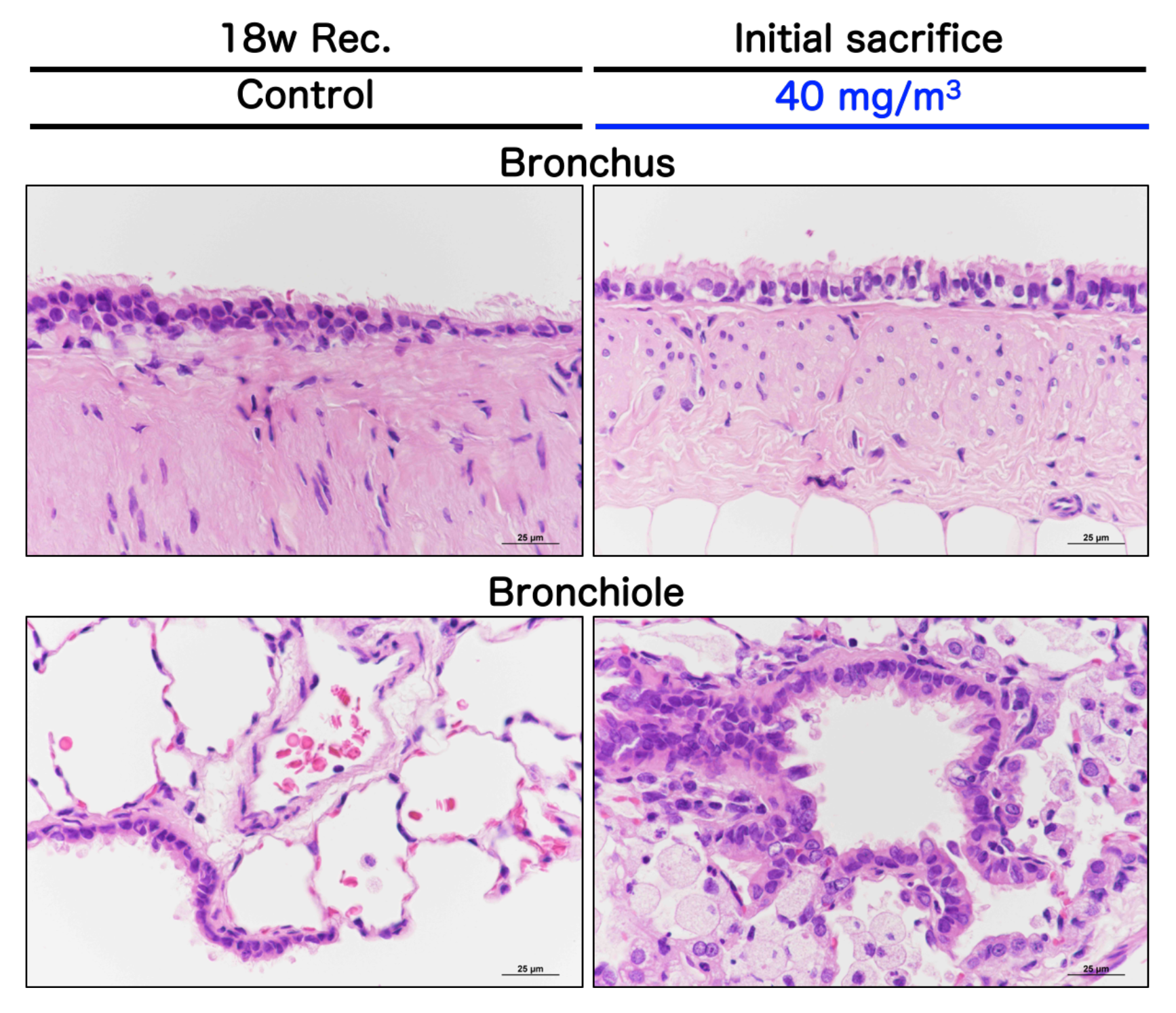

Supplement: Supplementary file 7 — Additional file 7: Figure S7. Representative histopathological photographs of the bronchus and bronchiole in the rat lung after repeated inhalation exposure to CWAAP-A (40 mg/m3). All data are the results of high-concentration intermittent inhalation study. [file 12931_2023_2355_MOESM7_ESM.tif]

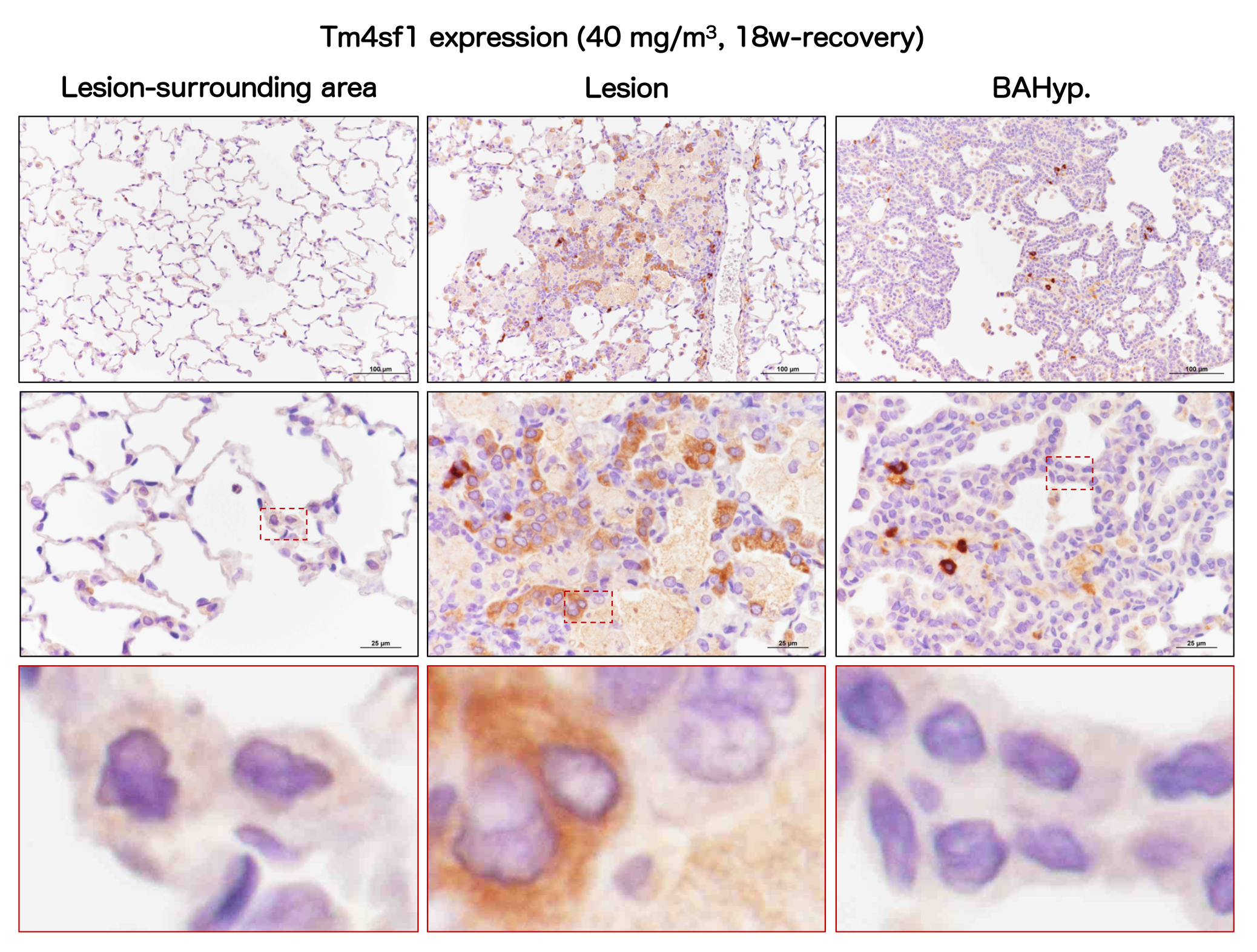

Supplement: Supplementary file 8 — Additional file 8: Figure S8. Representative images of Tm4sf1 expression in the rat lung after repeated inhalation exposure to 40 mg/m3 CWAAP-A. All data are the results of high-concentration intermittent inhalation study. [file 12931_2023_2355_MOESM8_ESM.tif]

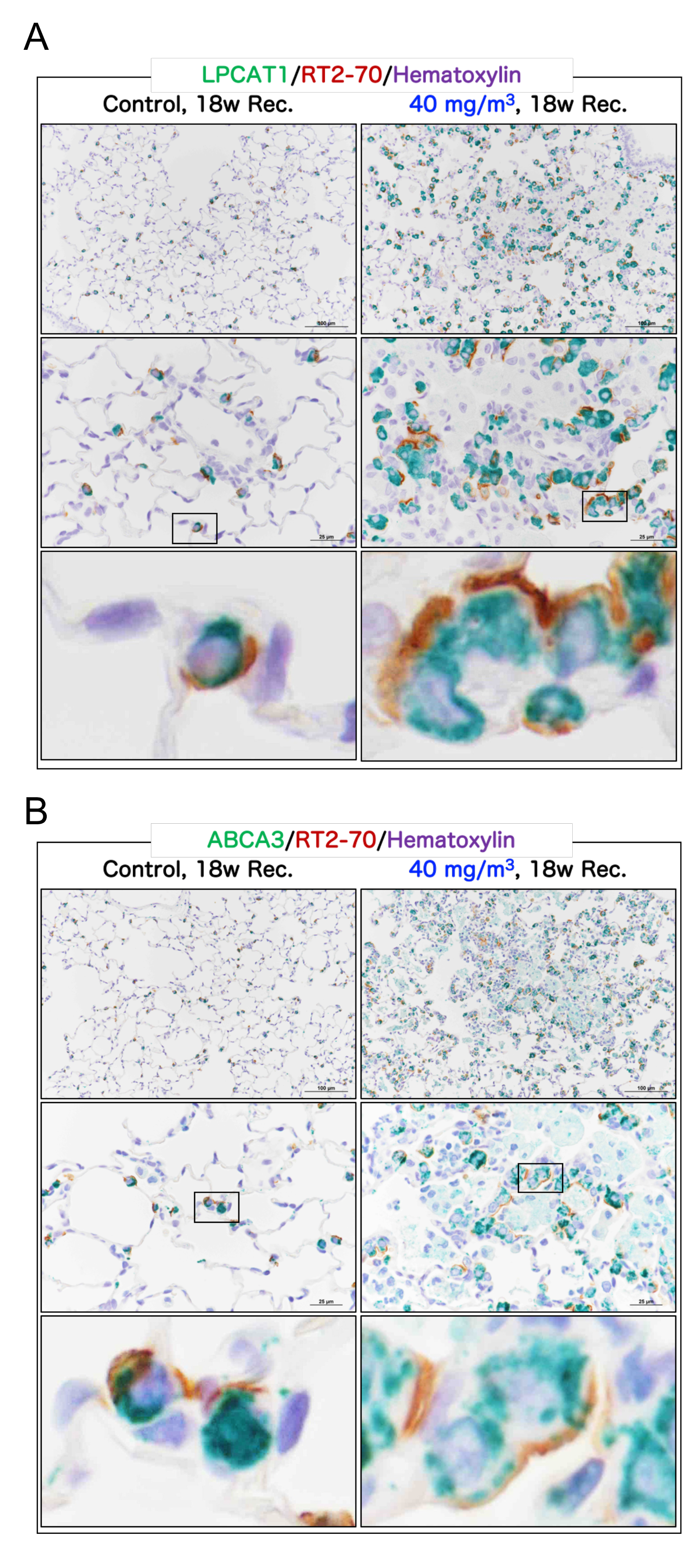

Supplement: Supplementary file 9 — Additional file 9: Figure S9. Figure S9. Representative images of the AEC2 markers co-staining. Representative images of the AEC2 membranous marker RT2-70 co-staining with AEC2 cytoplasmic markers LPCAT1 (A)and ABCA3 (B) in the rat lung. [file 12931_2023_2355_MOESM9_ESM.tif]

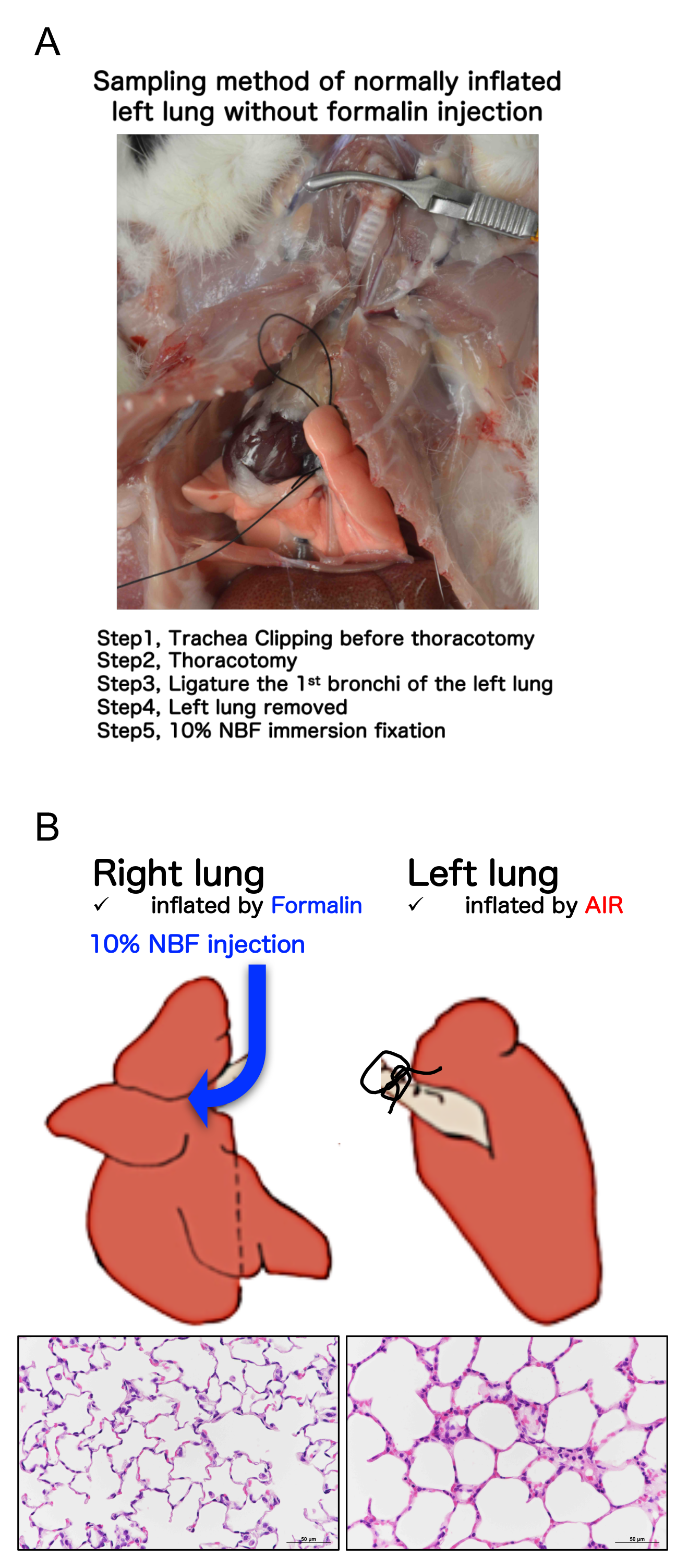

Supplement: Supplementary file 10 — Additional file 10: Figure S10. Sampling procedure of a single inhalation exposure study. Sampling procedure of the air inflated left lung without formalin injection (A) and comparative images of the right lung inflated by formalin and the left lung inflated by air (B). [file 12931_2023_2355_MOESM10_ESM.tif]

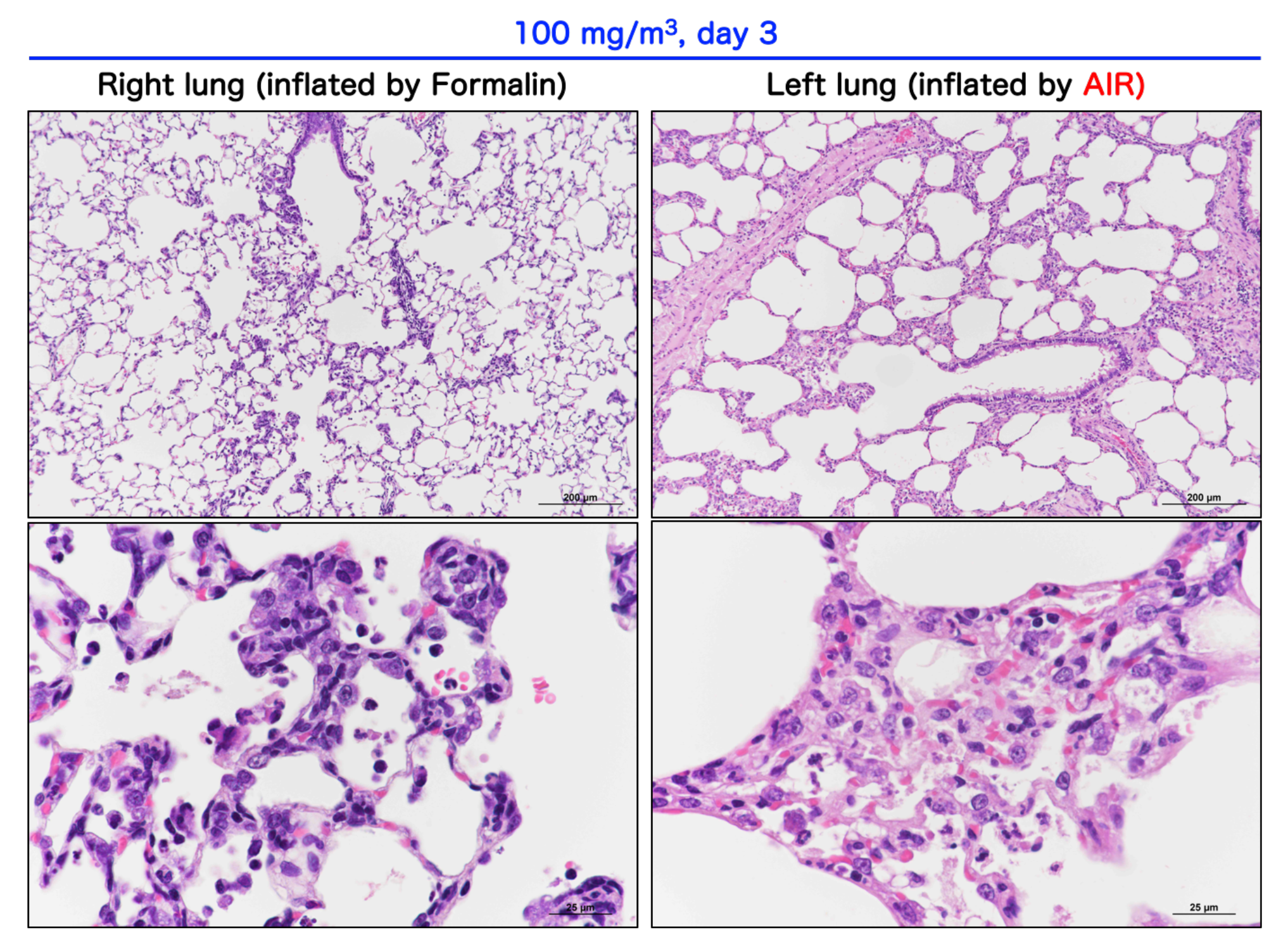

Supplement: Supplementary file 11 — Additional file 11: Figure S11. Representative lung histopathological images of a single inhalation exposure study. Representative histopathological images of the right lung inflated by formalin and the left lung inflated by air of a rat 3 days after exposure to 100 mg/m3: the right lung and the left lung are from the same animal. [file 12931_2023_2355_MOESM11_ESM.tif]

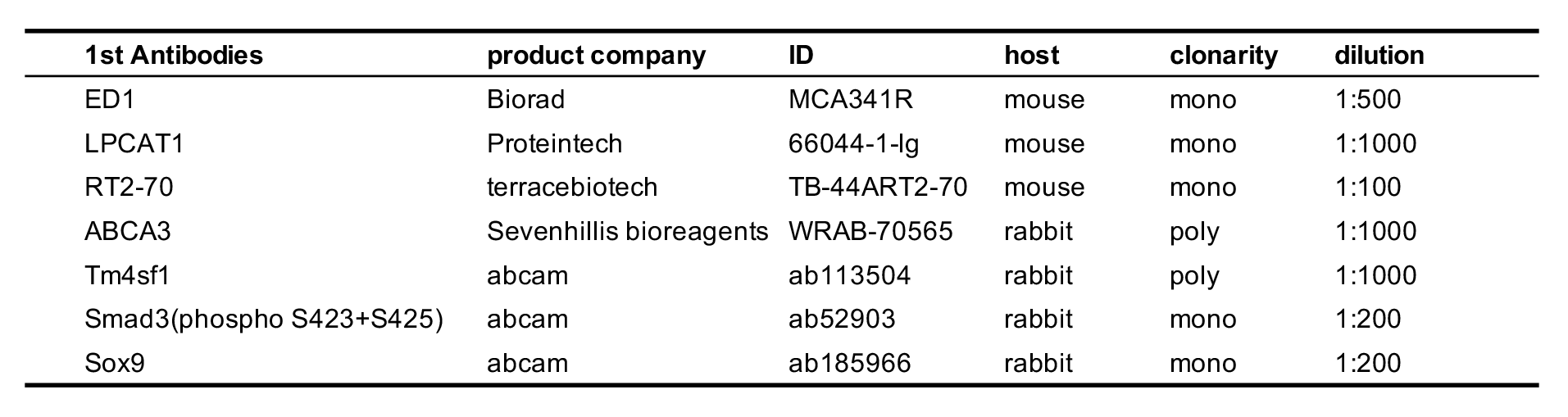

Supplement: Supplementary file 12 — Additional file 12: Table S1. List of primary antibodies used in this study. [file 12931_2023_2355_MOESM12_ESM.tif]

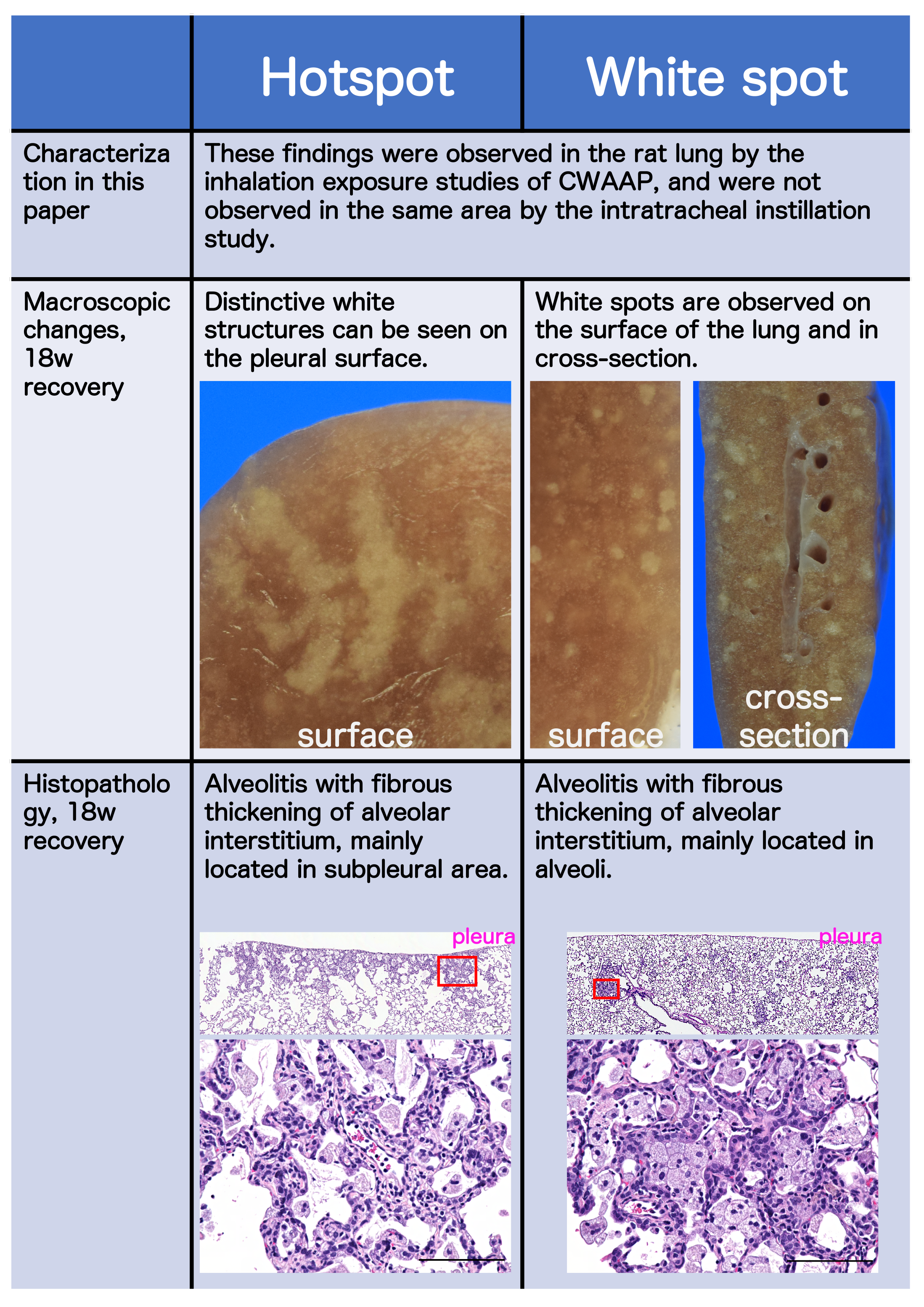

Supplement: Supplementary file 13 — Additional file 13: Table S2. Summary of differences between hotspot and white spot. [file 12931_2023_2355_MOESM13_ESM.tif]
