# Supplementary material for: Mechanisms of pulmonary disease in F344 rats after workplace-relevant inhalation exposure to cross-linked water-soluble acrylic acid polymers
Source: Respir Res. 2023 Feb 13;24:47. doi: 10.1186/s12931-023-02355-z (PMC9926550; doi:10.1186/s12931-023-02355-z)
Supplement: Supplementary file 2 — Additional file 2: Figure S2. The three animal experimental protocols used in this study. The high-concentration intermittent (repeated) inhalation exposure study (A), the high-concentration single inhalation study (B), and the repeated intratracheal instillation study (C). [file 12931_2023_2355_MOESM2_ESM.pdf]

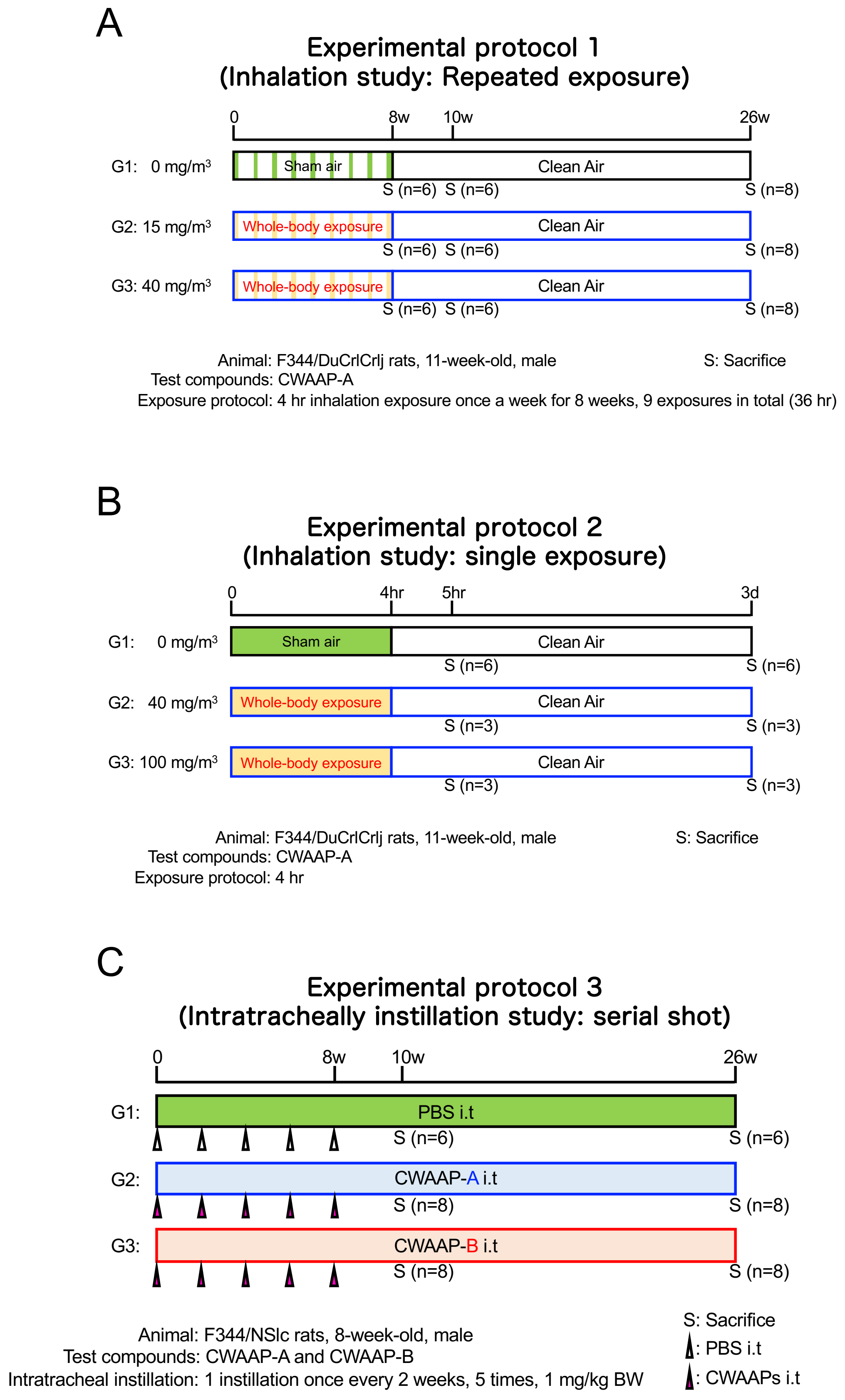
A
Experimental protocol 1
(Inhalation study: Repeated exposure)
0
8w
10w
26w
G1: 0 mg/m3
Sham air
Clean Air
S (n=6) S (n=6)
S (n=8)
G2: 15 mg/m3
Whole-body exposure
Clean Air
S (n=6) S (n=6)
S (n=8)
G3: 40 mg/m3
Whole-body exposure
Clean Air
S (n=6) S (n=6)
S (n=8)
Animal: F344/DuCrlCrlj rats, 11-week-old, male
S: Sacrifice
Test compounds: CWAAP-A
Exposure protocol: 4 hr inhalation exposure once a week for 8 weeks, 9 exposures in total (36 hr)
B
Experimental protocol 2
(Inhalation study: single exposure)
0
4hr
5hr
3d
G1: 0 mg/m3
Sham air
Clean Air
S (n=6)
S (n=6)
G2: 40 mg/m3
Whole-body exposure
Clean Air
S (n=3)
S (n=3)
G3: 100 mg/m3
Whole-body exposure
Clean Air
S (n=3)
S (n=3)
Animal: F344/DuCrlCrlj rats, 11-week-old, male
S: Sacrifice
Test compounds: CWAAP-A
Exposure protocol: 4 hr
C
Experimental protocol 3
(Intratracheally instillation study: serial shot)
0
8w
10w
26w
G1:
PBS i.t
S (n=6)
S (n=6)
G2:
CWAAP-A i.t
S (n=8)
S (n=8)
G3:
CWAAP-B i.t
S (n=8)
S (n=8)
S: Sacrifice
: PBS i.t
: CWAAPs i.t
Animal: F344/NSlc rats, 8-week-old, male
Test compounds: CWAAP-A and CWAAP-B
Intratracheal instillation: 1 instillation once every 2 weeks, 5 times, 1 mg/kg BW
